# Beneficial effects of recombinant CER-001 high-density lipoprotein infusion in sepsis: results from a bench to bedside translational research project

**DOI:** 10.1186/s12916-023-03057-5

**Published:** 2023-11-02

**Authors:** Alessandra Stasi, Marco Fiorentino, Rossana Franzin, Francesco Staffieri, Sabrina Carparelli, Rosa Losapio, Alberto Crovace, Luca Lacitignola, Maria Teresa Cimmarusti, Francesco Murgolo, Monica Stufano, Cesira Cafiero, Giuseppe Castellano, Fabio Sallustio, Chiara Ferrari, Mario Ribezzi, Nicola Brienza, Annalisa Schirinzi, Francesca Di Serio, Salvatore Grasso, Paola Pontrelli, Cyrille Tupin, Ronald Barbaras, Constance Keyserling-Peyrottes, Antonio Crovace, Loreto Gesualdo

**Affiliations:** 1 Nephrology, Dialysis and Transplantation Unit, Department of Precision and Regenerative Medicine and Ionian Area (DiMePRe-J), University of Bari, Bari, Italy; 2 Veterinary Surgery Unit, Department of Precision and Regenerative Medicine and Ionian Area (DiMePRe-J), University of Bari, Bari, Italy; 3 Department of Veterinary Medicine, University of Sassari, Sassari, Italy; 4 Division of Anesthesiology and Resuscitation, Department of Precision and Regenerative Medicine and Ionian Area (DiMePRe-J), University of Bari, Bari, Italy; 5 Department of Clinical Sciences and Community Health, University of Milan, Milan, Italy; 6 Department of Interdisciplinary Medicine-Intensive Care Unit Section, University of Bari, Bari, Italy; 7 Clinical Pathology Unit, University of Bari, Bari, Italy; 8 Abionyx Pharma, 31130 Balma, France

**Keywords:** ApoA-I complexes, Sepsis, Multi-organ dysfunction, Cytokine storm

## Abstract

**Background:**

Sepsis is characterized by a dysregulated immune response and metabolic alterations, including decreased high-density lipoprotein cholesterol (HDL-C) levels. HDL exhibits beneficial properties, such as lipopolysaccharides (LPS) scavenging, exerting anti-inflammatory effects and providing endothelial protection. We investigated the effects of CER-001, an engineered HDL-mimetic, in a swine model of LPS-induced acute kidney injury (AKI) and a Phase 2a clinical trial, aiming to better understand its molecular basis in systemic inflammation and renal function.

**Methods:**

We carried out a translational approach to study the effects of HDL administration on sepsis. Sterile systemic inflammation was induced in pigs by LPS infusion. Animals were randomized into LPS (*n* = 6), CER20 (single dose of CER-001 20 mg/kg; *n* = 6), and CER20 × 2 (two doses of CER-001 20 mg/kg; *n* = 6) groups. Survival rate, endothelial dysfunction biomarkers, pro-inflammatory mediators, LPS, and apolipoprotein A-I (ApoA-I) levels were assessed. Renal and liver histology and biochemistry were analyzed. Subsequently, we performed an open-label, randomized, dose-ranging (Phase 2a) study included 20 patients with sepsis due to intra-abdominal infection or urosepsis, randomized into Group A (conventional treatment, *n* = 5), Group B (CER-001 5 mg/kg BID, *n* = 5), Group C (CER-001 10 mg/kg BID, *n* = 5), and Group D (CER-001 20 mg/kg BID, *n* = 5). Primary outcomes were safety and efficacy in preventing AKI onset and severity; secondary outcomes include changes in inflammatory and endothelial dysfunction markers.

**Results:**

CER-001 increased median survival, reduced inflammatory mediators, complement activation, and endothelial dysfunction in endotoxemic pigs. It enhanced LPS elimination through the bile and preserved liver and renal parenchyma. In the clinical study, CER-001 was well-tolerated with no serious adverse events related to study treatment. Rapid ApoA-I normalization was associated with enhanced LPS removal and immunomodulation with improvement of clinical outcomes, independently of the type and gravity of the sepsis. CER-001-treated patients had reduced risk for the onset and progression to severe AKI (stage 2 or 3) and, in a subset of critically ill patients, a reduced need for organ support and shorter ICU length of stay.

**Conclusions:**

CER-001 shows promise as a therapeutic strategy for sepsis management, improving outcomes and mitigating inflammation and organ damage.

**Trial registration:**

The study was approved by the Agenzia Italiana del Farmaco (AIFA) and by the Local Ethic Committee (N° EUDRACT 2020–004202-60, Protocol CER-001- SEP_AKI_01) and was added to the EU Clinical Trials Register on January 13, 2021.

**Supplementary Information:**

The online version contains supplementary material available at 10.1186/s12916-023-03057-5.

## Background

Therapies to prevent organ dysfunction in sepsis and septic shock are limited [1]. During sepsis, early and appropriate antibiotic administration to counteract infection is the cornerstone of treatment; additionally, current therapeutic strategies include supportive therapy mainly based on hemodynamic stabilization and, when organ dysfunction occurs, organ support [2]. No specific treatments to prevent or recover renal injury in septic patients are available to date and renal replacement therapy (RRT) is required when renal function is severely compromised [3]. Moreover, the use of blood purification techniques, including high-volume hemofiltration, polymyxin B hemoperfusion, and cytokines absorber devices, has not shown significant benefits to support their routine use in clinical practice and they are not recommended by the Surviving Sepsis Campaign Guidelines [4–7].

The cytokine storm induced by the underlying infection can rapidly lead to multi-organ failure such as cardiovascular and respiratory systems, liver or kidney dysfunctions at advanced stages [8]. The cytokine storm is an overwhelming reaction of the innate immune system following a bacterial or viral infection, which induces significant impairment of lipid and lipoprotein homeostasis in particular the high-density lipoprotein (HDL) [9]. HDL complexes in addition to their well-characterized reverse cholesterol transport from peripheral cells to the liver, display anti-inflammatory, antioxidant, and antithrombotic properties [10]. They regulate the function and integrity of endothelial cells, interfering with inflammatory and apoptotic stimuli [11]. Furthermore, HDL plays an essential role in shaping immune response and may have a direct anti-infectious effect, thereby decreasing infection severity [9, 12, 13]. Low HDL-cholesterol (HDL-C) and apolipoprotein A-I (ApoA-I) levels during the initial phase of sepsis is strongly associated with 30-day mortality and prolonged ICU stay, suggesting the ability of HDL to reduce TNF-alpha production induced by LPS [14]. Recently, a prospective observational study including 205 septic patients admitted in ICU reported that an HDL-C level at ICU admission < 0.4 mmol/l was associated with increased mortality at day 28 [15]. In the case of viral infection, such as SARS-CoV-2 infection, a significant decrease in HDL-C and ApoA-I were reported [16], and low levels of ApoA-I were associated with a poor prognosis for recovery [17]. This phenomenon can be generalized to sepsis regardless of infection source [13–15]. Interestingly, a recent publication described ApoA-I as the only lipid biomarker associated with long-term survival (1 year) in patients with surgical sepsis [18]. Altogether, one can hypothesize that the rapid restoration of small functional HDL particles containing ApoA-I in critically ill patients could have beneficial effects.

Several experimental studies have been performed in pre-clinical models of sepsis. Reconstituted HDL (CSL-111) administration in a mouse model of sepsis by cecal ligation and puncture or intraperitoneal injection of *E. coli* or *Pseudomonas aeruginosa* pneumonia improved overall survival, reducing the inflammatory burden and decreasing bacterial count [19]. Similarly, the supplementation of a synthetic HDL (sHDL ETC-642) improved organ dysfunction and 7-day survival in septic mice, not only by neutralizing LPS, but also by a significant suppression of TLR4-NF-ĸB signaling and pro-inflammatory cytokine production [20]. To date, there have been no significant experiences with HDL administration in the clinical setting in sepsis. In humans, the effect of infused synthetic reconstituted HDL in volunteers challenged with a sub-pathologic dose of LPS showed a decrease of cytokines such as IL-6 [21, 22]. This observation was attributed mainly to the scavenger effect of the HDL [21, 22]. Cases of patients with SARS-CoV-2 infection admitted to the ICU receiving short-term treatment (q12h for 1–2 days) with a synthetic HDL composed of a human recombinant ApoA-I and phospholipids, CER-001, were reported [23, 24]. In all cases, CER-001 was able to rapidly decrease the level of different cytokines with commensurate improvement of the patients’ clinical conditions [23]. These observations suggest that ApoA-I-complexes could have effects other than a scavenger effect per se and that there is some rationale to treat other patients such as septic patients with ApoA-I-complexes. To this purpose, we first studied the in vitro effect of CER-001 on peripheral blood mononuclear cell (PBMC) and endothelial cells, and based on the results obtained, we tested the effect of CER-001 in a swine model of LPS-induced acute kidney injury (AKI) and in an open-label Phase 2a pilot clinical trial. Our hypothesis was that CER-001 could provide anti-inflammatory and endothelial-protective effects, preserving kidney and liver integrity and function, reducing the risk of AKI as well as improving clinical outcomes.

## Methods

### Animal model

The animal study was performed in domestic swine, after approval by the ethical committee of the Italian Ministry of Education, University and Research (MIUR) (Prot. n.1234/2020-PR). The number of animals was chosen for an appropriate analysis by calculating the number of subjects necessary, using the calculator program Anastat (http://www.anastats.fr/). The evaluation of six pigs for each group was made in relation to the data obtained in our previous studies published on pig models of acute kidney injury [25–30]. We performed the statistical analysis with a significance level of *p* = 0.05. Briefly, endotoxemia was induced by intravenous infusion of a saline solution containing 300 μg/kg of LPS (lipopolysaccharide membrane of Escherichia coli), left untreated, the swine will progress to LPS-induced AKI, as previously described [29]. The animal model utilized represents a sterile inflammation model induced by LPS administration.

The animals were randomized into three groups: LPS (endotoxemic pigs, *n* = 6), CER20 (endotoxemic pigs treated with a single dose of CER-001 [Abionyx Pharma, Toulouse, France] 20 mg/kg, a few minutes after the start of LPS infusion; *n* = 6), and CER20 × 2 (endotoxemic pigs treated with two doses of CER001 20 mg/kg a few minutes after the start of LPS infusion and 3 h later; *n* = 6).

For all CER-001 dosing, the drug product was thawed and then diluted with normal saline to a volume of 250 mL containing 20 mg/kg of CER-001, individualized for each animal based on weight, and administered over a period of 1 h using an infusion pump at a fixed rate of 250 mL/h. The LPS group received 250 ml of normal saline solution at the same infusion rate.

Since CER-001 is a negatively charged lipoprotein complex that mimics natural HDL, consisting of a combination of recombinant human apolipoprotein A-I (ApoA-I) and two phospholipids, the administered dose of CER-001 will reflect the concentration of human ApoA-I to be delivered in the animal model.

Surviving animals were sacrificed after approximately 24 h from LPS/saline infusion with an overdose of IV propofol, immediately followed by a 10-ml IV bolus of an oversaturated solution of potassium chloride (2 mEq/ml, Galenica Senese, srl, Italy).

#### Collection of samples

At sacrifice or earlier death, kidneys and livers were collected from all animals and processed using standard procedures as previously described [29]. Urine samples were collected via catheter from all animals and urinary output was recorded every hour. Swine sera were collected at baseline (T0; before LPS infusion), and at intermediate time points up to 24 h from an indwelling arterial blood catheter. Bile samples were collected from all animals at sacrifice. LPS was extracted from bile samples using the phenol-water extraction method [31], with an LPS extraction kit (Intron Biotechnology, Kyungki-Do, Korea) according to the manufacturer’s instructions.

Total protein extraction was performed from all bile samples [32, 33]. One thousand microliters of bile of each sample was centrifuged at 9000 × *g* for 3 min at 4 °C, and the supernatant containing the soluble proteins was used for assessment of ApoA-I levels.

#### Assessment of LPS and ApoA-I levels

The ApoA-I content of sera and bile samples was determined by ELISA assay (R&D Systems, Minneapolis MN, USA) as well as LPS (R&D Systems, Minneapolis MN, USA).

#### Assessment of pro-inflammatory cytokines and markers of endothelial dysfunction

Serum IL-6 and TNF-α levels were measured by ELISA (R&D Systems, Minneapolis MN, USA) as well as s-VCAM-1, s-ICAM-1, and MCP-1 (MyBioSource, San Diego CA, USA).

#### Assessment of the activity of classical, lectin, and alternative complement pathways

Complement function in swine sera was assessed using ELISA (WIESLAB® Complement System Screen COMPL 300, Euro-Diagnostica) as previously described [34].

#### Kidney and Liver function measurements

Serum/urine creatinine, serum/urine Kidney Injury Molecule-1 (KIM-1), and serum/urine Cystatin C measurements were performed with commercially available ELISA kits (MyBioSource, San Diego, USA) according to the manufacturer’s instructions. Liver function was assessed by serum measurements of alanine aminotransferase (ALT) enzyme with commercially available ELISA (MyBioSource, San Diego, USA).

#### Histological analysis of renal and hepatic tissue

Renal and hepatic tissues were processed for histologic staining [hematoxylin and eosin (HE) (Millipore Sigma)]. Digital slides were acquired and analyzed using the AperioScanScope CS2 device (Aperio, Vista, CA, USA) as previously described [26, 29]. HE staining was performed to evaluate histological injury in both kidneys and livers. Tubular and glomerular damage was scored semi-quantitatively by two blinded observers. The score index in each animal was expressed as a mean value of all scores obtained. Both tubular and glomerular pathological scores for each group were expressed as mean ± SEM. Hepatic injury was defined as the amount of destruction of hepatic lobules, infiltration of inflammatory cells, hemorrhage, and hepatocyte necrosis [35]. The score, from 1 through 4, was assessed using criteria from a previously published study [35]. Pathological score for each group was expressed as mean ± SEM.

#### Western blotting analysis

Liver tissues were homogenized and treated with RIPA lysis buffer (1 mM PMSF, 5 mM EDTA, 1 mM sodium orthovanadate, 150 mM sodium chloride, 8 μg/mL leupeptin, and 1.5% Nonidet P-40, and 20 mM Tris–HCl, pH 7.4) with phosphatase and protease inhibitors. The samples (30 μg of proteins) were separated in 4–15% polyacrylamide gel and then transferred to PVDF membrane (0.2 mM) by Trans-Blot Turbo (BioRad, Hercules, CA, USA). Nonspecific binding sites on the blots were blocked by incubation in 5% BSA for 1 h, and the membranes were then incubated overnight with primary antibodies and incubated with secondary antibodies for 1 h. Immune complexes were detected by the ECL chemiluminescence system (Amersham Pharmacia, Little Chalfont, UK), according to the manufacturer’s instructions. The primary antibodies used were anti-LPS (Abcam) and anti-β-actin antibody (1:20,000; Sigma). The secondary antibodies used were HRP-conjugated anti-rabbit (Abcam) and anti-mouse antibodies (Abcam). The chemiluminescent blots were acquired by Chemidoc and analyzed using ImageJ software. The protein expression levels were standardized relative to the level of β-actin.

#### Cell culture

Human umbilical vein endothelial cells (HUVEC, EC) were purchased from American Type Culture Collection (ATCC-LGC Standards S.r.l., Sesto San Giovanni, Milan, Italy). EC were maintained in their recommended medium, EndGro (Merck Millipore, Darmstadt, Germany). Peripheral blood mononuclear cells (PBMCs) were isolated by gradient centrifugation with the Ficoll-Hypaque method from buffy coats of healthy donors (selected from our research repository) as previously described [36]. PBMCs were maintained in their recommended media [36]. When cells became confluent, they were stimulated with LPS 0.3 μg/ml, 4 μg/ml (*E. coli* O111:B4, Sigma-Aldrich, Milan, Italy) and CER-001 50, 100, and 500 μg/ml for the indicated time period. PBMC culture supernatants were collected and analyzed by ELISA for TNF-α (R&D Systems, Minneapolis MN, USA).

#### MTT assay

EC and PBMCs were incubated with LPS and CER-001 for 24 h. Proliferation rate was measured by MTT Cell Proliferation Assay Kit, according to the manufacturer instructions (Sigma-Aldrich). Briefly, 3 × 104 cells/well were seeded in a 96-well plate, and then cells were treated with LPS and CER-001 as indicated. Absorbance at 570 nm was then measured by a spectrophotometer.

#### Immunophenotypic analysis

After stimulations, EC were permeabilized with IntraPrep kit (Instrumentation Laboratory) and incubated with unconjugated primary antibody p-ENOS (Abcam) for 25 min at 4 °C. Cells were then washed and labeled with secondary Antibody AlexaFluor 488 (Molecular Probes) for 25 min at 4 °C. Finally, cells were washed twice and resuspended in FACS buffer for acquisition. PBMCs were stained with the following monoclonal antibody, CD14 Monoclonal Antibody (61D3)-PE, (eBioscience™, Thermo Fisher Scientific, Italy), for 20 min in the dark at room temperature, washed twice, and resuspended in FACS buffer. Stained PBMCs were then acquired.

Data were obtained by using a FC500 (Beckman Coulter) flow cytometer and analyzed with Kaluza software. Three independent experiments were performed for both EC and PBMCs. The area of positivity was determined by using an isotype-matched mAb, and in total, 104 events for each sample were acquired.

#### Statistical analysis

Survival data were analyzed using a log-rank test for trend; values were censored for surviving animals using the time of sacrifice. LPS, cytokines (TNF-α, MCP-1, IL-6), endothelial markers (VCAM-1, ICAM-1), complement (classical, alternate, lectin pathways), kidney biomarkers in serum (sCR, sKIM-1, sCystatin C) and urine (uKIM-1, uCystatin C), and ALT were analyzed using two-way ANOVA with repeated measures, corrected for multiple comparison of pairwise treatment group differences using Tukey’s method. For these analyses, data was transformed to change from baseline, and the last observation was carried forward when necessary to handle any missing values. Urine output, tubular injury score, glomerular injury score, and hepatic injury score were analyzed using one-way ANOVA, corrected for multiple comparison of pairwise treatment group differences using Tukey’s method. For the ANOVA analyses, pairwise treatments were also tested without correction for multiple comparisons (Additional File 1: Table S1). For in vitro analysis, data were shown as mean ± standard deviation (SD) and compared with the Student *t* test. All analyses were performed by using GraphPad Prism 9.2.0 (GraphPad software, Inc., San Diego, CA, USA).

#### Clinical trial design and setting

We performed an open-label, randomized, dose-ranging (Phase 2a) study including patients with sepsis due to intra-abdominal cavity infection or urosepsis, admitted at the intensive care units (ICUs) and the Sub-intensive Nephrology Unit at Azienda Ospedaliero-Universitaria Policlinico, Bari, Italy. The study was approved by the Agenzia Italiana del Farmaco (AIFA) and by the Local Ethic Committee (“A RAndomized pilot study comparing short-term CER-001 infusions at different doses to prevent sepsis-induced acute kidney injury, RACERS study”, N° EUDRACT 2020–004202-60, Protocol CER-001-SEP_AKI_01). All enrolled patients or legally authorized representatives provided written informed consent. A total of twenty patients were enrolled between June 2021 and September 2022 with the last follow-up visit occurring in October 2022.

#### Study population/participants

Adults aged 18 years or older with a diagnosis of sepsis were eligible to participate in the study if they met the following main inclusion criteria: (1) sepsis sustained by Gram-negative bacteria receiving antibiotic treatment; (2) met Sepsis 3 criteria [37]; (3) endotoxin activity assay (EEA™, Spectral Medical, Toronto, Canada) > 0.60; and (4) signed and dated informed consent provided by the patient or by a legal representative. Patients were excluded if they had conditions which either precluded the use of CER-001 or prevented the evaluation of CER-001’s effect on kidney or other organ systems.

#### Treatment allocation and study interventions

Twenty patients meeting the eligibility criteria, with a signed and dated an Ethical Committee (EC)-approved informed consent form, were randomized and assigned in a 1:1:1:1 ratio to one of four treatment groups, as shown in Additional File 2: Fig. S1. All patients received any and all necessary conventional (non-experimental) therapy (antibiotic treatments, sedation, pain relief, organ support, etc.), modulated according to the clinical conditions. Group A received no experimental treatment. Group B patients received CER-001 5 mg/kg BID; Group C patients received CER-001 10 mg/kg BID; and Group D patients received CER-001 20 mg/kg BID. CER-001 treatment was administered twice a day on days 1, 2, 3, and 6 and each dose was preceded with a dose of antihistamine to avoid any potential infusion reactions, which have been previously reported [38, 39].

Clinical and laboratory data were collected during each study visit, planned at treatment start (day 1), day 2, day 3, day 6, and day 9. Samples were drawn prior to the first dose on days 1, 2, 3, and 6. A final visit was performed on day 30. In addition to daily routine laboratory assessment performed at the hospital laboratory, biological samples (serum, plasma, and urine) were collected and adequately stored for each study visit to perform additional inflammatory cytokines and biomarkers included in the study protocol. Additional exploratory biomarkers were also tested on these banked specimens, which the patients had consented to prior to enrollment. Biomarker and cytokine testing was performed at the Research Laboratory at Nephrology Unit. Serum levels of LPS, IL-6, IL-8, MCP-1, TNF-α, soluble triggering receptor expressed on myeloid cells-1 (sTREM-1), s-VCAM-1, and s-ICAM-1 were measured by ELISA (R&D Systems, Minneapolis MN, USA).

#### Study objectives

The primary endpoint of the study was to determine an optimal dose of CER-001 in combination with standard of care in septic patients based on safety. In addition, the study aims to analyze whether CER-001 treatment had an effect on AKI onset and severity according to KDIGO criteria [40]. Secondary endpoints included the change from baseline (the last measurement taken prior to dosing on day 1) to days 3, 6, and 9 for endotoxin and IL-6 levels, SOFA score, and other key inflammatory and endothelial dysfunction markers. Data on mortality at day 30 were also reported and analyzed.

#### Statistical analysis

We did not perform a formal calculation of sample size since the present randomized trial was designed as a safety and dose-ranging study and the sample size was based on feasibility only. Comparisons between treatment and control groups were performed using the appropriate statistical tests: all continuous variables were compared by analysis of variance (ANOVA) or non-parametric equivalents, as appropriate. For most parameters, changes from baseline (day 1) to days 3, 6, and 9 were compared and graphically represented. Proportion of patients with each AKI stage and the mortality rate were calculated for each group. A *p*-value of less than 0.05 was considered statistically significant. No adjustments for multiple testing were made in this pilot study.

## Results

### In vitro effect of the ApoA-I complexes (CER-001)

We first analyzed the in vitro effect of CER-001 on both the endothelial integrity and its LPS scavenger effects on human PBMC. In our in vitro model, using human vascular endothelial cells, endothelial nitric oxide synthase (eNOS) phosphorylation and activation is altered by LPS and upregulated by CER-001 (Additional File 2: Fig. S2 A-B). In addition, CER-001 modulates the response of PBMC, decreasing mCD14 expression and TNF-α secretion both markers of LPS innate immunity system stimulation (Additional File 2: Fig. S2 C-E).

### ApoA-I complexes (CER-001) increased survival rate and dramatically decreased systemic inflammation and endothelial dysfunction in a swine model of sepsis

**Fig. 1:**
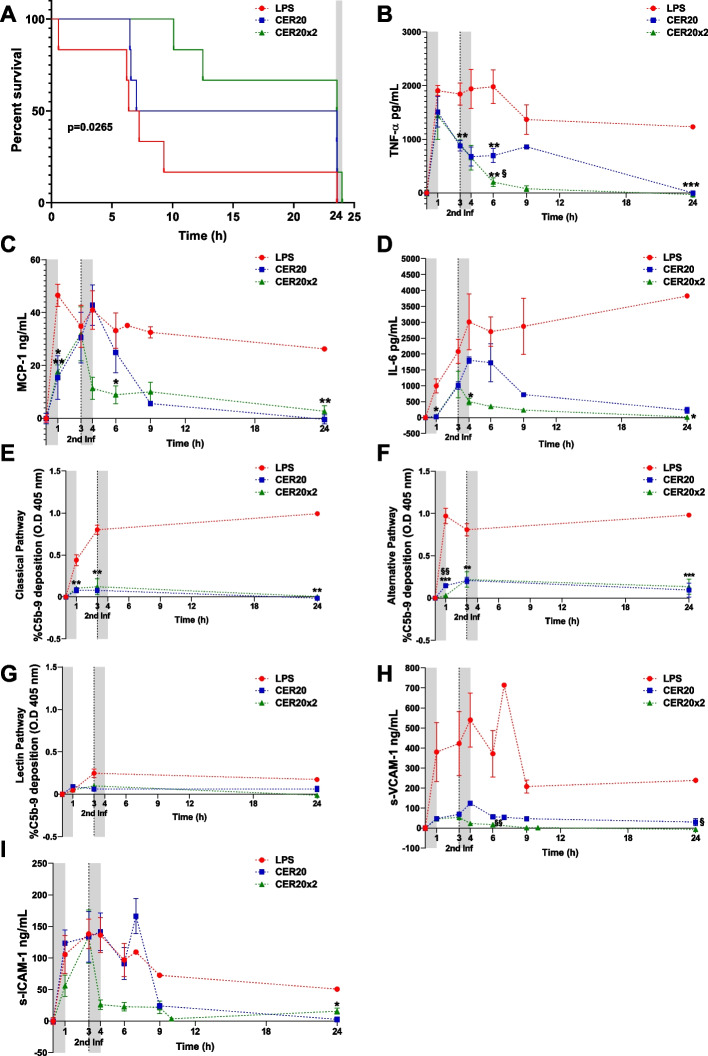
Improvement of survival rate and decrease of systemic pro-inflammatory response and endothelial dysfunction in a swine model of LPS-induced AKI. All animals received an infusion of LPS 300 µg/kg (T0) without (control group) or with infusion of CER-001 at 20 mg/kg (CER20 group) followed for half of this group by a second infusion at 3 h of 20 mg/kg of CER-001 (CER20 × 2 group). At T24, surviving animals were sacrificed (gray band). **A** Survival curve of pigs upon challenge with LPS and CER-001 infusions. A significant statistical treatment trend was observed by log-rank, in the three groups (*p* = 0.0265 log-rank trend test, *n* = 6). **B**–**D** Serum levels of TNF-α, MCP-1, and IL-6 were measured by ELISA assay (*n* = 6 independent samples per time point and group). **E**–**G** Systemic complement activation was measured by Wieslab assay (*n* = 6 independent samples per time point and group). **H**, **I** Serum levels of VCAM-1 and ICAM-1 were measured by ELISA assay (*n* = 6 independent samples per time point and group). In each graph, the gray bands show two infusions (0–1 h and 3–4 h) of saline or CER-001 with flow rates of 250 ml/h. Results are presented as mean ± SEM. Significant differences were assessed using a two-way ANOVA for repeated measures with Tukey correction for multiple comparisons (*p < 0.05 **p < 0.005, ***p < 0.0005 vs the LPS group; §*p* < 0.05; §§*p* < 0.005, §§§*p* < 0.0005 vs the CER20 group). Details of differences between Tukey correction and uncorrected Fisher’s LSD for significant comparisons are listed in Additional File 1: Table S1

Domestic swine (*Sus scrofa domesticus*) were infused with 300 µg/kg of LPS without (control group) or with infusion of CER-001 at 20 mg/kg (CER20 group) followed for half of this group by a second infusion at 3 h of 20 mg/kg of CER-001 (CER20 × 2 group). Untreated pigs were highly susceptible to LPS challenge and usually succumbed before completing the study protocol with a survival rate of approximately 16.7%. CER-001 treatment significantly increased median survival of endotoxemic pigs by 50 and 66.7%, in CER20 and CER20 × 2 groups, respectively (Fig. 1A).

Then, we assessed a representative set of pro-inflammatory molecules. TNF-α, rapidly rises, followed in time by increasing MCP-1 and IL-6 in endotoxemic animals (LPS group, Fig. 1B–D). This increase was significantly diminished by CER-001 (T24, CER20 vs LPS group: TNF-α, *p* = 0.0004) with a more potent effect after 2 doses of CER-001 (T24, CER20 × 2 vs LPS group, TNF-α, *p* < 0.0001; MCP-1, *p* = 0.0009; IL-6, *p* = 0.0086). In addition, CER-001 treatments significantly inhibited the systemic complement activation cascade, resulting in a notable decrease of the original activating pathway, including the classical, alternative, and lectin pathways, with the latter being less stimulated by LPS infusion (Fig. 1E–G).

Finally, we evaluated systemic biomarkers of endothelial dysfunction. High levels of s-VCAM-1 and s-ICAM-1 were measured in endotoxemic pigs after LPS infusion (Fig. 1H, I). CER-001 infusion ameliorated systemic endothelial dysfunction by reducing VCAM-1 (Fig. 1H) and ICAM-1 (Fig. 1I) serum levels in both treated groups, with an increased effect of the two doses of ApoA-I complexes as emphasized at T6 time points.

### ApoA-I complexes (CER-001) treatment prevented liver and renal dysfunction

**Fig. 2:**
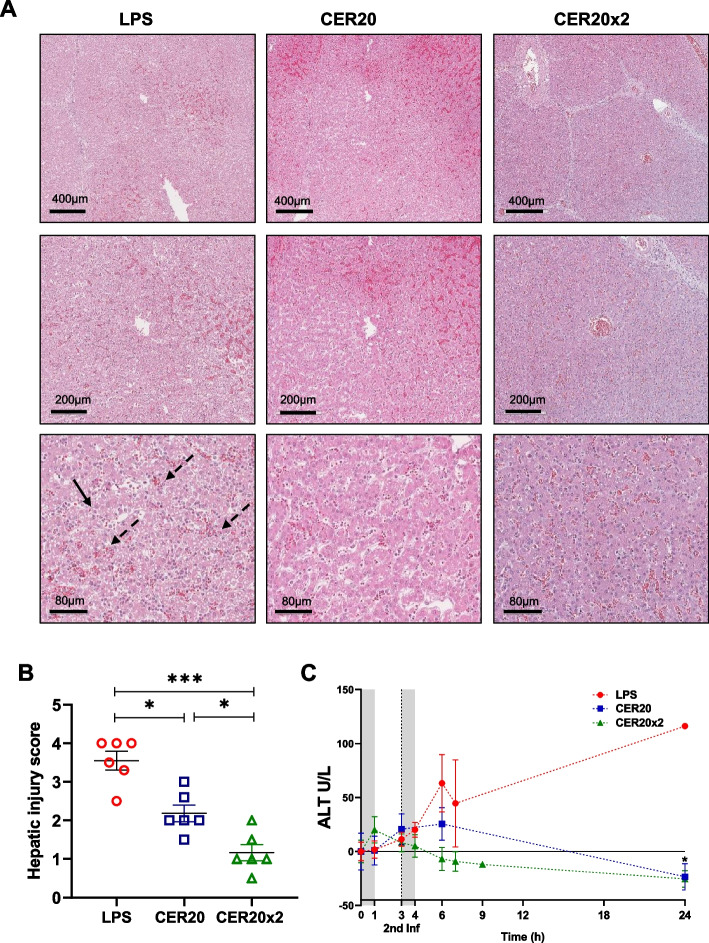
Improvement of liver dysfunction following ApoA-I-complexes (CER-001) infusions. **A** Top panel shows representative H&E staining of hepatic tissue of LPS and treated groups (*n* = 6 independent pigs per group). Liver sections (upper panel, scale bar = 400 μm; middle panel, scale bar = 200 μm; lower panel, scale bar = 80 μm). Hepatic injury was defined as the amount of destruction of hepatic lobules (black arrow) and infiltration of inflammatory cells (black dashed arrow). **B** Liver injury was calculated from whole images of stained liver, as calculated from five randomly selected fields per sample (*n* = 6 independent pigs per group) and scored from 1 through 4 according to % area of involvement per HPF. Results are presented as mean ± SEM. Significant differences were assessed by one-way ANOVA with Tukey correction (*p < 0.05, **p < 0.005, ***p < 0.0005). **C** Serum levels of ALT enzyme were measured by ELISA assay (*n* = 6 independent samples per time point and group). The gray bands show two infusions (0–1 h and 3–4 h) of saline or CER-001 with flow rates 250 ml/h. Results are presented as mean ± SEM. Significant differences were assessed using a two-way ANOVA for repeated measures with Tukey correction (n.s.: *p* > 0.05, *p < 0.05, **p < 0.005, ***p < 0.0005 vs the LPS group; §*p* < 0.05; §§*p* < 0.005, §§§*p* < 0.0005 vs the CER20 group). Details of differences between corrected and uncorrected comparisons are listed in Additional File 1: Table S1

In endotoxemic pigs, we observed early manifestations of hepatic dysfunction which included histological changes such as hepatic areas with micro-vacuolization and infiltrating inflammatory cells and increased ALT levels (Fig. 2A–C). The hepatocytes appeared swollen, and the hepatic cords were disorganized. In this setting, the hepatic damage was resolved by CER-001 infusion (as measured by the modest increase in ALT and a statistically significant decrease of liver histology score), underscoring the protection that this drug mediates at the hepatic level, thereby preserving its physiological function.

**Fig. 3:**
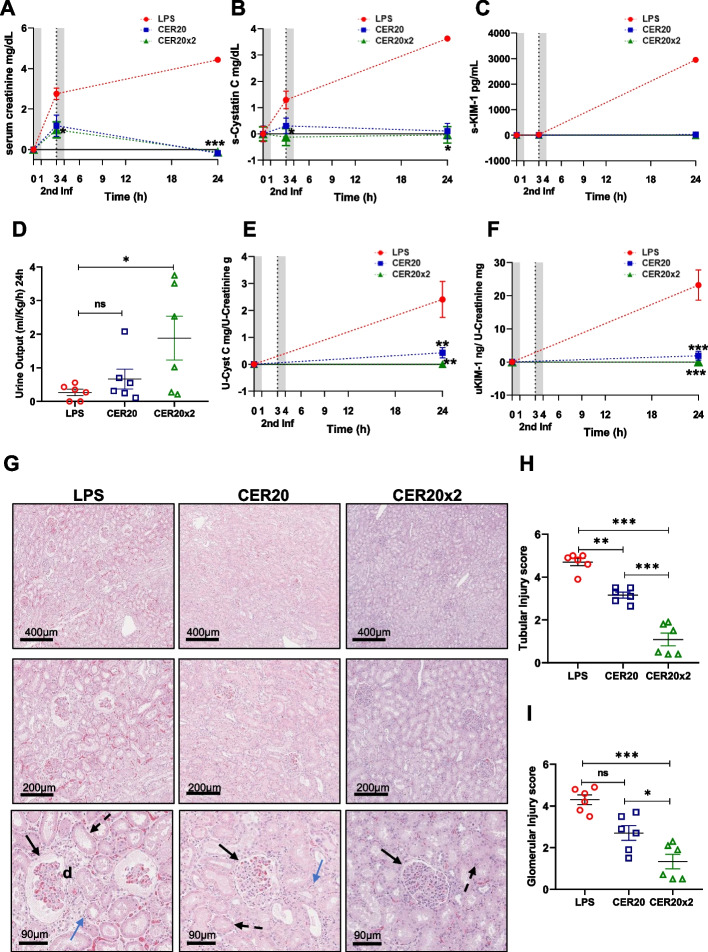
Improvement of renal dysfunction following ApoA-I-complexes (CER-001) infusions. **A**–**F** sCr, sCystatin C, sKIM-1, UCr, U-Cys, and U-KIM-1 levels were measured by ELISA assay (*n* = 6 independent samples per time point and group); urinary output (ml/kg/h) was recorded for each animal. The gray bands show two infusions (0–1 h and 3–4 h) of saline or CER-001 with flow rates 250 ml/h. Results are presented as mean ± SEM. Significant differences were assessed using a two-way ANOVA for repeated measures with Tukey correction (n.s.: *p* > 0.05, *p < 0.05, **p < 0.005, ***p < 0.0005 vs the LPS group; §*p* < 0.05; §§*p* < 0.005, §§§*p* < 0.0005 vs the CER20 group). **G**, H & E staining was performed on paraffin kidney sections. Renal sections (upper panel, scale bar = 400 μm; middle panel, scale bar = 200 μm; lower panel, scale bar = 80 μm). The kidney desquamation and vacuolization of proximal tubular epithelial cells (zoomed image, 80 µm, black dashed arrow), fibrin deposition and loss of capillaries in numerous glomeruli, Bowman’s capsule expansion (zoomed image, 80 μm, black arrow), and interstitial inflammatory infiltrate (zoomed image, 80 μm, blue arrow) were observed by H&E staining. **H**, **I** Tubular and glomerular pathological score was obtained as described in the “Methods” section (*n* = 6 for each group). Results are presented as mean ± SEM. Significant differences were assessed by one-way ANOVA with Tukey correction (*p < 0.05 **p < 0.005, ***p < 0.0005). Details of differences between corrected and uncorrected comparisons are listed in Additional File 1: Table S1

Kidney injury in the swine model was assessed by a time-dependent increase of serum creatinine with significant reduction in urinary output (mL/kg/h) (Fig. 3A, D) compared to basal level (T0). Moreover, the expression of biomarkers of tubular damage, Cystatin C and KIM-1, both in serum (Fig. 3B, C) and urine samples (Fig. 3E, F) were increased compared with the basal level (T0), whereas CER-001 treatment maintained creatinine level, urine output, the expression of serum/urine Cystatin C, and KIM-1 to the median baseline level (T0) as compared to control animals. This renal damage was highlighted by significant morphological changes in renal parenchyma, including tubular vacuolization, epithelial flattening, necrosis, infiltration of inflammatory cells, and marked fibrin deposition and reduced number of capillaries in numerous glomeruli (Fig. 3G, LPS group). Those observations were reflected by the histopathological score which shows significantly less kidney damage in treated groups especially after two doses of ApoA-I complexes (Fig. 3H, I).

### Excretion of LPS into the bile via CER-001 transport

**Fig. 4:**
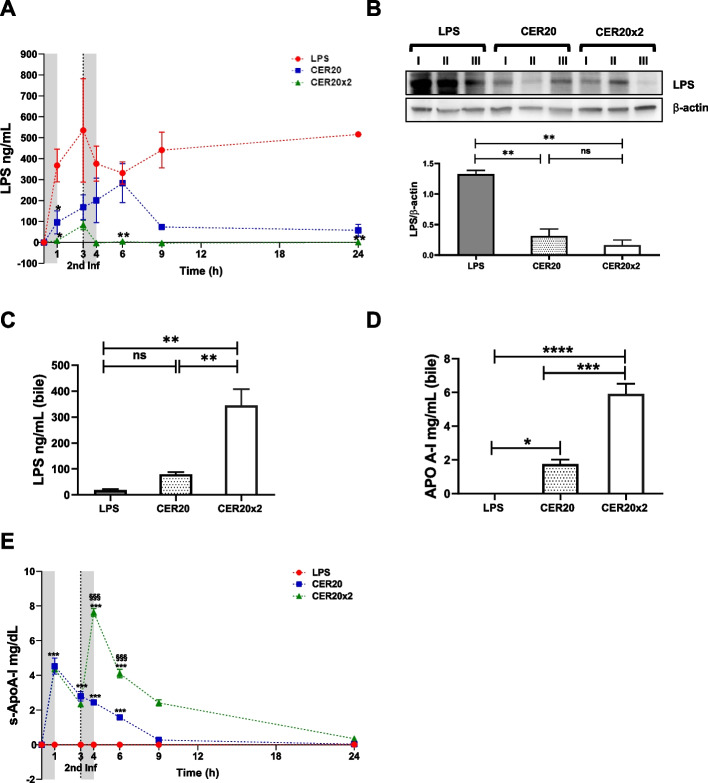
Excretion of LPS into the bile via CER-001 transport. **A** Serum levels of LPS were measured by ELISA assay (*n* = 6 independent samples per time point and group). **B** Representative western blot and densitometric analysis of LPS and β-actin protein expression (*n* = 6 per group). **C** Endotoxin levels in bile samples were dosed by ELISA assay. **D** Human ApoA-I levels in bile samples were evaluated by ELISA assay. **E** The serum human ApoA-I for CER-001 was determined at different time points by ELISA assay. The gray bands show two infusions (0–1 h and 3–4 h) of saline or CER-001 with flow rates 250 ml/h. The data are presented as the mean ± SEM. Statistically significant differences were assessed by one-way ANOVA with Tukey correction (*p < 0.05, **p < 0.005, ***p < 0.0005 vs the LPS group; §*p* < 0.05; §§*p* < 0.005, §§§*p* < 0.0005 vs the CER20 group)

Consistent with CER-001’s positive effects, we observed that serum LPS levels were reduced in treated animals (Fig. 4A) and the effects were especially evident after the second infusion of CER-001 (T6, CER20 × 2 vs LPS, *p* = 0.0015) with a sustained effect up to 24 h post-infection in both treated groups. Then, we assessed the levels of LPS in both liver tissue and bile samples. The amount of LPS measured in the liver was higher for LPS group than for both CER-001 treated groups whereas, as shown in Fig. 4, a dose-dependent increase of endotoxin in the bile of CER-001-treated septic pigs was detected (Fig. 4C). Interestingly, a parallel dose-dependent increase of human ApoA-I was found in bile samples (Fig. 4D). Moreover, a significant increase in serum ApoA-I levels was observed in animals treated with CER-001 (Fig. 4E).

### Pilot study of infused ApoA-I complexes in a heterogeneous population of sepsis patients

We conducted an exploratory Phase 2a pilot study comparing a short course of CER-001 infusions, added to standard therapeutic treatments, to standard treatments alone, in septic patients (RACERS study, EUDRACT number 2020–004202-60, Protocol CER-001-SEP_AKI_01). Table 1 summarizes the main baseline characteristics between treated and control groups. Patients were generally similar between the two groups, with a small imbalance in the underlying infection source (SOC patients were predominantly urinary tract infections), which did not affect the other baseline clinical parameters. Patients were enrolled in an early phase of sepsis, as the median time of enrollment from hospital admission was 2 days. The severity of the disease as well as organ dysfunction scores did not differ between SOC and treatment group.

**Table 1:** Baseline clinical and demographic characteristics of RACERS participants

Intervention	SOC group	CER-001 group	*p*-value
	***n* = 5**	***n* = 15**	
Age (years), mean (SD)	59 (10.29)	56.6 (18.63)	0.789
Male gender, *n* (%)	2 (40%)	4 (26.4%)	0.573
**Site of infection/sepsis, *n*(%)**			
Urine	4 (80%)	7 (46.7%)	
Intra-abdominal	0	6 (40%)	0.238
Other	1 (20%)	2 (13.3%)	
Septic shock, *n* (%)	1 (20%)	6 (40%)	0.795
**Hospital unit, *n*(%)**			
Intensive care unit	2 (40%)	7 (46.7%)	0.795
Nephrology	3 (60%)	8 (53.3%)	
SOFA score, mean (SD)	6.6 (6.07)	6.07 (4.62)	0.830
SAPS II score, mean (SD)	31.8 (11.9)	27.6 (14.2)	0.569
Immunocompromised patients, *n* (%)	1 (20%)	1 (13.3%)	0.717
Time for enrollment from hospital admission, median (IQR)	0 (0–26)	2 (1-6)	1
Mean arterial pressure (MAP), mean (SD)	85.8 (9.73)	81 (14.22)	0.495
Vasopressors use, *n* (%)	1 (20%)	6 (40%)	0.573
Vasopressors dose (mcg/kg/min), mean (SD)^a^	0.2 (0.1)	0.17 (0.1)	0.630
Mechanical ventilation, *n*(%)	2 (40%)	6 (40%)	1
Acute kidney injury (AKI), *n* (%)	3 (60%)	8 (53.3%)	0.795
Renal replacement therapy (RRT), *n* (%)	1 (20%)	2 (13.3%)	0.717

*Legend*. *SD* standard deviation, *ICU* intensive care unit, *ARDS* acute respiratory distress syndrome, *SAPS II* Simplified Acute Physiology Score II, *SOFA* Sequential Organ Failure Assessment

^a^Values calculated only on ICU patients on vasopressors at treatment start

Nine patients were enrolled in the ICU, 6 of them developed post-surgical intra-abdominal sepsis with blood culture and/or intra-abdominal drainage fluid culture positive for *Klebsiella pneumoniae* (3 patients), *Pseudomonas aeruginosa* (3 patients), and *Acinetobacter baumanni* (1 patient). Two ICU patients were enrolled following sepsis/septic shock with positive blood culture for *Klebsiella pneumoniae* and *Acinetobacter baumanni*, while one ICU patient was enrolled with urosepsis and positive blood culture for *Bacteroides fragilis*. Eleven patients were enrolled in the Nephrology Unit, mainly related to urosepsis with blood and/or urine culture positive for *Klebsiella pneumoniae* (1 patient), *Proteus mirabilis* (2 patients), while microbiological results were negative in 7 patients, although they were characterized by laboratory and radiological signs of pyelonephritis and urosepsis. Another patient was enrolled in the Nephrology Unit due to central venous catheter infection sustained by *Klebsiella pneumoniae* and *Pseudomonas aeruginosa*. All patients were screened for high levels of endotoxin activity to confirm the presence of Gram-negative infection prior to study enrollment and in advance of culture results.

CER-001 was well-tolerated compared to SOC under the conditions of this study. There were no serious adverse events attributed to the use of CER-001 during the study (Additional File 3: Table S2).

### ApoA-I complexes decreased inflammation and endothelial dysfunction in septic patients

**Fig. 5:**
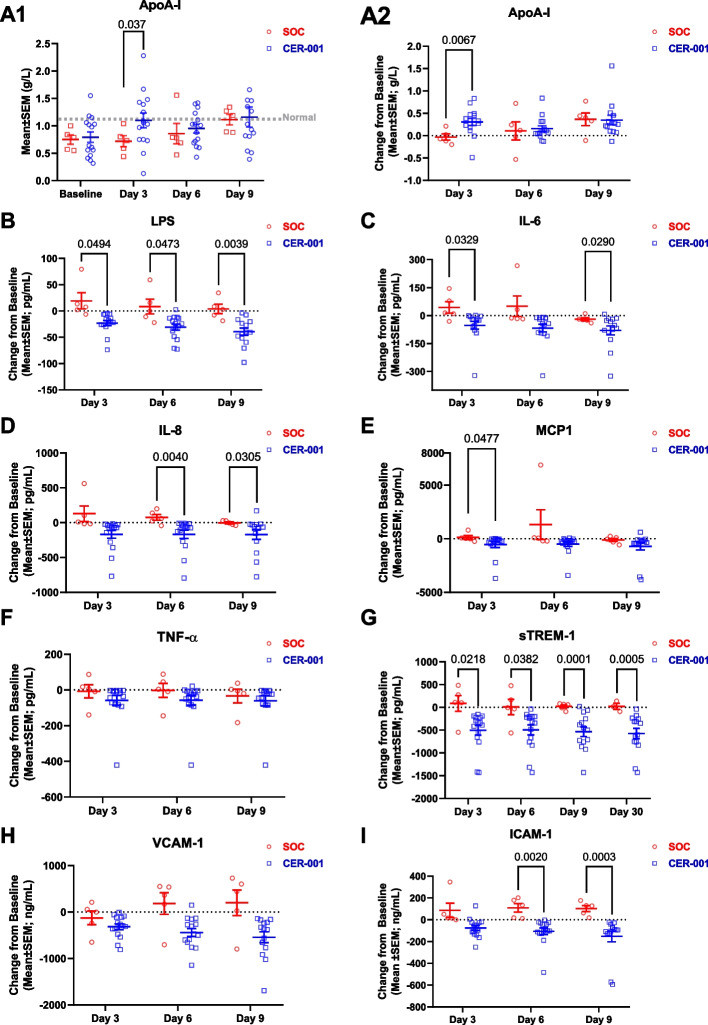
Effect of CER-001 on ApoA-I level, LPS removal, and inflammatory response in the pilot study RACERS. **A**–**I** Serum levels of ApoA-I, LPS, IL-6, IL-8, MCP1, TNF-α, sTREM-1, VCAM, and ICAM were measured using ELISA kits. Data for panels A2–I are represented as changes from baseline (day 1 pre-dose) values. Treatment differences were assessed by using a mixed model ANOVA for values on days 3, 6, and 9 with a significance level of *α* = 0.05; significant pairwise comparisons are shown. No adjustments were made for multiple comparisons. Abbreviations: ApoA-I apolipoprotein A-I; ICAM endothelial intercellular adhesion molecule; IL-6 interleukin 6; IL-8 interleukin 8; IL-10 interleukin 10; LPS lipopolysaccharides; MCP-1 monocyte chemoattractant protein-1; TNF-α tumor necrosis factor α; sTREM-1 soluble triggering receptor expressed on myeloid cells 1; VCAM vascular cell adhesion molecule

Serum ApoA-I levels increased rapidly with CER-001 treatment, while the restoration of ApoA-I was delayed in the SOC group (Fig. 5A1, A2). Consistent with animal data and the observed ApoA-I increase, treatment with CER-001 significantly reduced bloodstream concentrations of LPS compared to SOC subjects and these decreases were sustained even after the administration of CER-001 ended (Fig. 5B).

Consequent to the scavenging of LPS, our data show that CER-001 treatment is associated with rapid and marked decreases in pro-inflammatory cytokines relative to SOC alone (Fig. 5C–F), most notably in IL-6, IL-8, and MCP-1. In addition, soluble triggering receptor expressed on myeloid cells-1 (sTREM-1) rapidly decreases with CER-001 treatment and remains low and stable through at least 30 days (Fig. 5G).

Finally, CER-001 treatment provided endothelial protection, as evidenced by markedly decreased values of circulating s-VCAM-1 and s-ICAM-1 in treated patients relative to increased values in patients on SOC alone (Fig. 5H, I). Individual CER-001 groups were also evaluated versus SOC (Additional File 2: Fig. S3).

### Effect of ApoA-I complexes on renal and liver dysfunction in septic patients

**Fig. 6:**
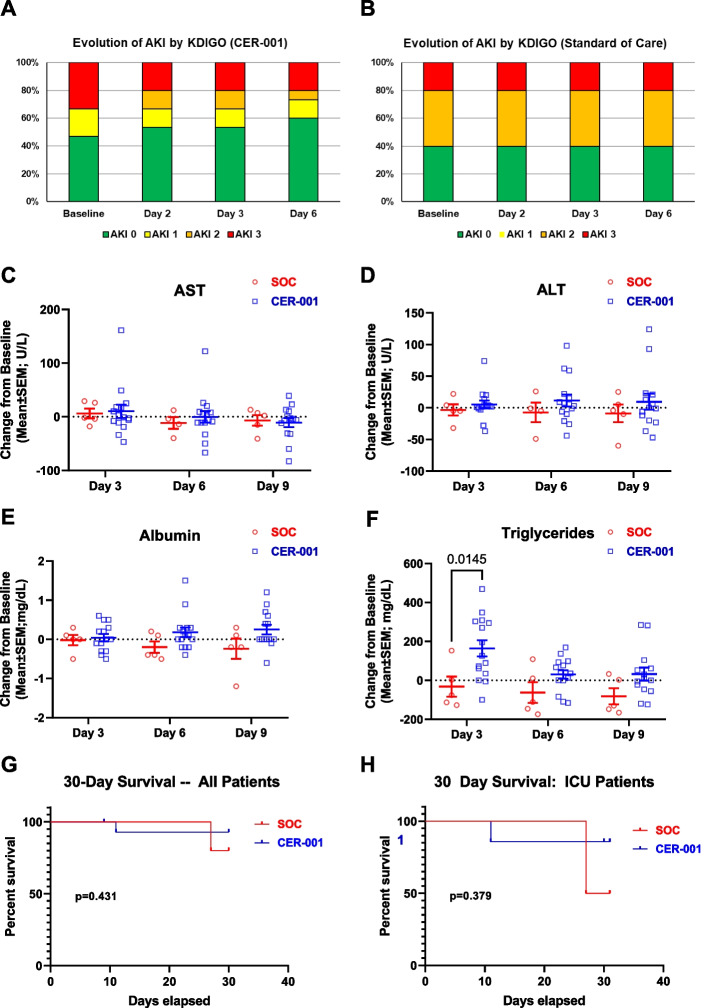
Effect of CER-001 on organ systems and survival. **A** Evolution of AKI among patients randomized to CER-001, according to KDIGO criteria from baseline (day 1 pre-dose) to day 6. **B** AKI stages among patients randomized to the Standard of Care group, according to KDIGO criteria from baseline (day 1 pre-dose) to day 6. **C**–**F** Serum levels of AST, ALT and albumin measured by the local hospital lab. Data are represented as changes from baseline (day 1 pre-dose) values. Treatment differences were assessed by using a mixed model ANOVA for values on days 3, 6, and 9 with a significance level of *α* = 0.05; significant pairwise comparisons are shown. No adjustments were made for multiple comparisons. **G**, **H** 30-day survival curves for all patients and for ICU patients. Treatment differences were assessed using the log-rank test with a significance level of *α* = 0.05. Abbreviations: AKI acute kidney injury; ALT Alanine aminotransferase; AST Aspartate aminotransferase

We analyzed the effects of CER-001 treatment on limiting renal dysfunction. Overall, we reported a low risk for the onset and/or progression to moderate to severe AKI (Stage 2 or 3) through day 6 among CER-001-treated patients (26.6%) relative to SOC, in which about 60% of patients presented such conditions (Fig. 6A, B). We also analyzed the effects of CER-001 on liver function. As shown in Fig. 6C, D, we did not observe significant alteration of liver enzymes (AST, ALT) among CER-001 subjects; two patients in the treated group presented non-clinically significant, transient increases in AST or ALT. Conversely, we observed a general increase of albumin levels in the CER-001-treated group compared to SOC (Fig. 6E). A transient but significant increase in triglycerides levels was documented on day 3 in 7 patients treated with CER-001 (Fig. 6F).

### Effect of ApoA-I complexes on clinical outcomes

We examined whether the observed beneficial biological effects of CER-001 would impact survival. Figure 6G shows 30-day survival in all patients. One patient died in the CER-001 group (6.7%) and one patient died on standard treatment (20%) (Fig. 6H). In the subset of ICU patients, the observed 30-day death rates were 50% on SOC and 14.2% with CER-001 treatment.

We focused on a subset of critically ill patients enrolled in the ICU to analyze the main clinical outcomes (7 treated patients; 2 SOC subjects, Additional File 4: Table S3). We observed a reduced length of ICU stay among patients in the treatment group (mean days of ICU stay 23.2 vs 28.5) (Additional File 2: Fig. S4 A-B). In addition, the daily average mean arterial pressure (MAP) during the study period improved with CER-001 treatment as compared to SOC (Additional File 2: Fig. S4 C; *p* = 0.036 on day 6) even though the use of vasopressors was similar for the two groups (Additional File 2: Fig. S4 D). Days on mechanical ventilation were lower among CER-001-treated patients (mean 17.1 vs 26.5; Additional File 2: Fig. S4 E). Similarly, the number of days on dialysis was also lower among CER-001 treated patients (mean 3.7 vs 11.0; Additional File 2: Fig. S4 F). Finally, the need for any form of organ support (a composite endpoint including mechanical ventilation, dialysis, use of vasopressors and survival) during the 30-day study period was lower in the treated subjects (mean days alive and without organ support 5.9 vs 2.0; Additional File 2: Fig. S4 G).

## Discussion

This translational study examined the potential role of an engineered HDL-mimetic, CER-001, as a novel adjuvant therapy in sepsis. Our findings demonstrated significant scavenger effects of CER-001 on endotoxins through the bile, as well as its ability to modulate the cytokine storm, reduce endothelial dysfunction, and prevent organ damage. Notably, we conducted a proof-of-concept clinical study that confirmed the pleiotropic effects of CER-001 in septic patients, in reducing renal damage, the need for organ support and ICU stay, emphasizing the potential clinical significance of this therapeutic approach.

Current treatment protocols for septic patients are based on hemodynamic resuscitation, supportive therapy, and adequate antibiotic therapy [41]. However, in most critically ill patients, these measures are not enough to prevent sepsis-related organ dysfunction and the onset of AKI. Although the hemodynamic instability and kidney hypoperfusion are historically considered the main mechanism involved, multiple experimental studies have highlighted the detrimental effects of an uncontrolled systemic inflammatory response in the pathophysiology of sepsis-associated AKI [42, 43]. Additionally, this systemic inflammation is correlated with alterations in lipid and lipoprotein metabolism [9]. Indeed, clinical observational studies report that decreased amount of HDL-C in septic patients is positively correlated with the severity of the illness [44]. Besides their well-documented role in reverse cholesterol transport (RCT), HDLs are involved in modulation of endothelial dysfunction, oxidative stress, inflammation, immune response, and coagulation activity [45]. Previous studies using reconstituted HDL and HDL-mimetic peptides have shown their ability to reduce mortality and attenuate the pro-inflammatory response in endotoxemia and sepsis [43]. Interestingly, the compelling evidence demonstrating the remarkable capacity of CER-001, an HDL-mimetic lipoprotein containing recombinant human ApoA-I, to enhance cholesterol mobilization, promote reverse cholesterol transport, and exhibit in vitro anti-inflammatory properties in an animal model of atherosclerosis suggests a strong potential for its efficacy in the treatment of septic conditions [46].

In the present animal study, we demonstrate the targeted anti-inflammatory effects of CER-001 at the renal and hepatic levels as well as at systemic circulatory system. The potential mechanism of action is due to CER-001’s capacity on the one hand to decrease the inflammatory cytokines and on the other hand to counteract the action of LPS. Our pre-clinical results suggest that CER-001 enhances the transport of LPS to the liver and promotes its elimination into the bile, indirectly attenuating inflammation. Interestingly, LPS and ApoA-I levels increased in bile samples of CER-001-treated animals. In addition, the pharmacokinetics of the human ApoA-I measured in the swine sera of both treated groups was consistent with the decreased amount of LPS, mediated by CER-001 treatment.

Furthermore, we demonstrated the ability of CER-001 to ameliorate systemic endothelial dysfunction and induced an increase in average survival time. The subsequent activation of endothelial cells upregulates the expression of different adhesion molecules, such as s-ICAM-1, s-VCAM-1, and selectins, that enhance leukocyte migration and homing, amplifying innate and adaptative immune response [47]. Accordingly, we observed an increased release of soluble adhesion molecules, s-ICAM-1 and s-VCAM-1, in sera of endotoxemic pigs and septic patients. Moreover, our analyses demonstrated a dose-dependent inhibition of these mediators in CER-001-treated subjects. In addition, our data showed that CER-001 treatment reduced serum levels of pro-inflammatory cytokines, such as TNF-α, MCP-1, and IL-6, both in endotoxemic pigs and in CER-001-treated patients. This observation is encouraging and suggests that CER-001, by decreasing LPS levels and interacting with the immune system through ApoA-I, limits the cytokine cascade and provides endothelial protection. Remarkably, we also found decreased levels of sTREM-1 after CER-001 treatment in septic patients that could be associated with increased survival and ameliorated prognosis. sTREM-1 has been suggested as a strong predictor of poor prognosis and mortality in septic patients [48]. Persistently high sTREM-1 levels during the first days following ICU admission are associated with mortality in human septic shock [49]. Moreover, CER-001 treatment reduced complement activation, demonstrating effectiveness in regulating the innate immune response.

Since HDL-C levels significantly declined in severe sepsis and septic shock, several studies have evaluated the impact of HDL in worsening renal function as a potential predictive biomarker and therapeutic target. In line with these findings, our study highlights the critical role of CER-001 in recovering renal function and urine output in endotoxemic animals. These results are consistent with the clinical data that reported a low risk for the onset and/or progression to severe AKI (Stage 2 or 3) among CER-001-treated patients.

Liver dysfunction is a fairly common manifestation during sepsis and it is strongly associated with mortality [50]. The relationship between HDL-C and sepsis-associated liver dysfunction has been previously studied, as HDL-C levels are typically lower in patients with liver dysfunction, although HDL-C concentration did not correlate with hepatic dysfunction markers and overall mortality [51]. The pathophysiology of such relationship is poorly understood; while it could be suspected to be a decreased hepatic synthesis of HDL during sepsis, an increased HDL elimination should be considered [51]. In this study, we observed early manifestations of hepatic dysfunction which included increased ALT levels and histological changes. Remarkably, hepatic damage was resolved by CER-001 infusion, underscoring the hepatoprotective effects of this drug, thereby preserving its physiological function.

Finally, the strong observed effect of CER-001 on the cytokine cascade and endothelial dysfunction led us to evaluate its potential impact on clinical outcomes, although this small pilot clinical trial could only detect trends in such clinical parameters. Interestingly, the observed mortality rates for SOC are consistent with a recent publication of a meta-analysis examining mortality from sepsis in hospitalized patients (30.1%) and in the subset of ICU-treated patients (40.4%) in the European region (as defined by the WHO) [52, 53]. We reported a more rapid improvement of clinical conditions among patients who received CER-001, as evidenced by a reduced length of ICU stay and need for organ support during the 30-day study period, as compared to SOC patients. These results need to be confirmed by a larger clinical trial examining these endpoints more rigorously.

Together, the present study has several strengths. We demonstrated the safety and efficacy of a novel HDL-mimetic compound in sepsis using a translational approach. We provide valuable insights into the pleiotropic effects of HDL in a heterogeneous population of septic patients. However, we acknowledge some limitations in our research. The application of pre-clinical results to the clinical setting is always questionable due to the difficulty of simulating the complexities of human disease. Another limitation is the open-label study design of the clinical study. Additionally, the sample size of our clinical study is limited as it was a proof-of-concept study, warranting caution in the interpretation of the results.

The findings of our research have profound implications for the management of sepsis and other critical illnesses characterized by inflammation and organ failure. Beyond sepsis, the multifaceted therapeutic potential of CER-001 may extend to other high-mortality clinical indications marked by inflammation. Notably, CER-001’s pleiotropic effects could hold promise in mitigating the severity and mortality of COVID-19 infection, as well as other critical illnesses such as acute respiratory distress syndrome (ARDS) and severe bacterial and viral infections. The ability to counteract inflammation, modulate the immune response, and protect endothelial cells makes it a potential therapeutic option worth exploring further. Considering the economic burden and high mortality associated with sepsis, the novel therapeutic approach related by CER-001 offers hope for addressing unmet medical needs in critical care.

Therefore, based on the positive outcomes of the human proof-of-concept trial, we will design and conduct a larger phase 2b/3 study. Phase 2b will be designed to further refine the dose and dose regimen. Phase 3 will allow us to demonstrate the clinical benefits of this promising therapy for patients, and to determine whether there is an economic impact for the treatment of sepsis.

## Conclusions

In conclusion, our translational study provides compelling evidence for the potential role of CER-001, an HDL-mimetic, as a novel adjuvant therapy in sepsis. The significant scavenger effects on endotoxins, modulation of the cytokine storm, reduction of endothelial dysfunction, and prevention of organ damage underscore the therapeutic promise of CER-001. Importantly, the pleiotropic effects of CER-001 in restoring normal ApoA-I levels in septic patients seems to translate into positive clinical outcomes in the sepsis population studied, independently of the type and gravity of the sepsis. Altogether, CER-001 has the potential to be a gamechanger for critical illnesses marked by inflammation and organ failure across different high mortality clinical indications which continue to have high unmet medical needs.

### Supplementary Information

**Additional file 1:** **Table S1.** Statistical differences between Tukey correction and Uncorrected Fisher's LSD for significant comparisons in pre-clinical model.

**Additional file 2:** **Figure S1.** RACERS Trial design. **Figure S2.** In vitro effects of CER-001 on LPS-treated endothelial cells and PBMCs. A-B. Cultured endothelial cells were stimulated with LPS at 0.3 ug/ml and/or CER-001 at 50 and 500 ug/ml for 60. FACS (Fluorescence Activated Cell Sorting) showed a strong decrease of eNOS (phospho S1177) (p-ENOS) after 60’ of LPS stimulation compared to basal and VEGF as a positive control. CER-001 supplementation at 500 ug/ml, completely reversed LPS effects. A. Representative data from one out of a total of three experiments are shown. B. Histograms indicate p-ENOS expression levels. C-E. PBMC from 3 different healthy donors were stimulated with LPS at 0.3 ug/ml and/or CER-001 at 50 and 500 ug/ml for 24 h. C-D FACS showed a strong upregulation of CD14 surface expression 24 h following LPS stimulation. PBMCs treated with LPS and CER-001 in combination maintained CD14 expression at basal level. C. One representative of three independent experiments is shown. D. Histograms indicate CD14 expression levels. E. PBMC culture supernatants were analyzed by ELISA. After 24 h from LPS stimulation, PBMCs increased TNF-α synthesis. Stimulation of PBMCs with CER-001 at 50 and 500 ug/ml alone did not influence TNF-α production. The addition of CER-001, both at 50 and 500 ug/ml, in culture media of LPS-activated PBMCs reverted LPS effects. A-E Data are representative of three independent experiments. Data are shown as mean ± standard deviation (SD) and compared with the Student-t test. **Figure S3.** Effect of CER-001 on ApoA-I level, LPS removal and inflammatory response in the pilot study RACERS presented by individual treatment groups. A-I. Serum levels of ApoA-I, LPS, IL-6, IL-8, MCP1, TNF-α, sTREM-1, VCAM and ICAM were measured using ELISA kits. Data for panels A2-I are represented as changes from baseline (Day 1 pre-dose) values. Treatment differences were assessed by using a mixed model ANOVA for values on Days 3, 6 and 9 with a significance level of α = 0.05; significant pairwise comparisons are shown. No adjustments were made for multiple comparisons. Abbreviations: ApoA-I apolipoprotein A-I; ICAM endothelial intercellular adhesion molecule; IL-6 Interleukin 6; IL-8 Interleukin 8; IL-10 Interleukin 10; LPS lipopolysaccharides; MCP-1 Monocyte Chemoattractant Protein-1; TNF-α Tumor Necrosis factor α; sTREM-1 soluble Triggering receptor expressed on myeloid cells 1; VCAM vascular cell adhesion molecule. **Figure S4.** Effect of CER-001 on length of stay and need for organ support in ICU patients. A-B. Days in ICU presented as survival curve and as mean/SEM number of days. C. Daily average MAP. Data is presented as change from Baseline (24 h preceding first dose) and analyzed using a mixed model ANOVA for values on Days 1 through 9 with a significance level of α = 0.05; significant pairwise comparisons are shown. No adjustments were made for multiple comparisons. D. Number of days with vasopressor use. E. Number of days with mechanical ventilation use. F. Number of days on dialysis. G-H Days alive without organ support presented as survival curve and as mean/SEM number of days.

**Additional file 3:** **Table S2.** Adverse events among participants randomized reported in the pilot clinical study.

**Additional file 4:** **Table S3.** Main clinical outcomes at 30 days of ICU patients of RACERS participants.

**Additional file 5.** 

## Data Availability

The datasets used and/or analyzed during the current study are available from the corresponding author on reasonable request.

## Abbreviations

AIFA: Agenzia Italiana del Farmaco
AKI: Acute kidney injury
ALT: Alanine aminotransferase
ANOVA: Analysis of variance
ApoA-I: Apolipoprotein A-I
ARDS: Acute respiratory distress syndrome
AST: Aspartate aminotransferase
eNOS: Endothelial nitric oxide synthase
HDL: High-density lipoprotein
HDL-C: HDL-cholesterol
HE: Hematoxylin and eosin
HUVEC: Human umbilical vein endothelial cells
ICUs: Intensive care units
KIM-1: Kidney Injury Molecule-1
LPS: Lipopolysaccharides
PBMC: Peripheral blood mononuclear cell
RCT: Reverse cholesterol transport
RRT: Renal replacement therapy
SD: Standard deviation
SOC: Standard of Care
sTREM-1: Soluble triggering receptor expressed on myeloid cells-1

## References

[1] Cavaillon JM, Singer M, Skirecki T (2020). Sepsis therapies: learning from 30 years of failure of translational research to propose new leads. EMBO Mol Med.

[2] Evans L, Rhodes A, Alhazzani W, Antonelli M, Coopersmith CM, French C (2021). Surviving sepsis campaign: international guidelines for management of sepsis and septic shock 2021. Intensive Care Med.

[3] Bellomo R, Kellum JA, Ronco C, Wald R, Martensson J, Maiden M (2017). Acute kidney injury in sepsis. Intensive Care Med.

[4] Cruz DN, Antonelli M, Fumagalli R, Foltran F, Brienza N, Donati A (2009). Early use of polymyxin B hemoperfusion in abdominal septic shock: the EUPHAS randomized controlled trial. JAMA.

[5] Dellinger RP, Bagshaw SM, Antonelli M, Foster DM, Klein DJ, Marshall JC (2018). Effect of targeted polymyxin B hemoperfusion on 28-day mortality in patients with septic shock and elevated endotoxin level: The EUPHRATES Randomized Clinical Trial. JAMA.

[6] Joannes-Boyau O, Honoré PM, Perez P, Bagshaw SM, Grand H, Canivet JL (2013). High-volume versus standard-volume haemofiltration for septic shock patients with acute kidney injury (IVOIRE study): a multicentre randomized controlled trial. Intensive Care Med.

[7] Monard C, Bianchi N, Poli E, Altarelli M, Debonneville A, Oddo M (2023). Cytokine hemoadsorption with CytoSorb(®) in post-cardiac arrest syndrome, a pilot randomized controlled trial. Crit Care.

[8] Jarczak D, Nierhaus A (2022). Cytokine storm-definition, causes, and implications. Int J Mol Sci..

[9] Khovidhunkit W, Kim MS, Memon RA, Shigenaga JK, Moser AH, Feingold KR (2004). Effects of infection and inflammation on lipid and lipoprotein metabolism: mechanisms and consequences to the host. J Lipid Res.

[10] Morin EE, Guo L, Schwendeman A, Li XA (2015). HDL in sepsis - risk factor and therapeutic approach. Front Pharmacol.

[11] Tanaka S, Couret D, Tran-Dinh A, Duranteau J, Montravers P, Schwendeman A (2020). High-density lipoproteins during sepsis: from bench to bedside. Crit Care.

[12] Feingold KR, Grunfeld C (2012). Lipids: a key player in the battle between the host and microorganisms. J Lipid Res.

[13] Barlage S, Gnewuch C, Liebisch G, Wolf Z, Audebert FX, Glück T (2009). Changes in HDL-associated apolipoproteins relate to mortality in human sepsis and correlate to monocyte and platelet activation. Intensive Care Med.

[14] Chien JY, Jerng JS, Yu CJ, Yang PC (2005). Low serum level of high-density lipoprotein cholesterol is a poor prognostic factor for severe sepsis. Crit Care Med.

[15] Tanaka S, Stern J, Bouzid D, Robert T, Dehoux M, Snauwaert A (2021). Relationship between lipoprotein concentrations and short-term and 1-year mortality in intensive care unit septic patients: results from the HIGHSEPS study. Ann Intensive Care.

[16] Begue F, Tanaka S, Mouktadi Z, Rondeau P, Veeren B, Diotel N (2021). Altered high-density lipoprotein composition and functions during severe COVID-19. Sci Rep.

[17] Ulloque-Badaracco JR, Hernandez-Bustamante EA, Herrera-Añazco P, Benites-Zapata VA (2021). Prognostic value of apolipoproteins in COVID-19 patients: a systematic review and meta-analysis. Travel Med Infect Dis.

[18] Guirgis FW, Leeuwenburgh C, Moldawer L, Ghita G, Black LP, Henson M (2021). Lipid and lipoprotein predictors of functional outcomes and long-term mortality after surgical sepsis. Ann Intensive Care.

[19] Tanaka S, Genève C, Zappella N, Yong-Sang J, Planesse C, Louedec L (2020). Reconstituted high-density lipoprotein therapy improves survival in mouse models of sepsis. Anesthesiology.

[20] Guo L, Morin EE, Yu M, Mei L, Fawaz MV, Wang Q (2022). Replenishing HDL with synthetic HDL has multiple protective effects against sepsis in mice. Sci Signal..

[21] Pajkrt D, Doran JE, Koster F, Lerch PG, Arnet B, van der Poll T (1996). Antiinflammatory effects of reconstituted high-density lipoprotein during human endotoxemia. J Exp Med.

[22] Wurfel MM, Kunitake ST, Lichenstein H, Kane JP, Wright SD (1994). Lipopolysaccharide (LPS)-binding protein is carried on lipoproteins and acts as a cofactor in the neutralization of LPS. J Exp Med.

[23] Faguer S, Del Bello A, Danet C, Renaudineau Y, Izopet J, Kamar N (2022). Apolipoprotein-A-I for severe COVID-19-induced hyperinflammatory states: a prospective case study. Front Pharmacol.

[24] Tanaka S, Begue F, Veeren B, Tran-Dinh A, Robert T, Tashk P (2022). First Recombinant high-density lipoprotein particles administration in a severe ICU COVID-19 patient, a multi-omics exploratory investigation. Biomedicines..

[25] Castellano G, Stasi A, Intini A, Gigante M, Di Palma AM, Divella C (2014). Endothelial dysfunction and renal fibrosis in endotoxemia-induced oliguric kidney injury: possible role of LPS-binding protein. Crit Care.

[26] Stasi A, Franzin R, Divella C, Sallustio F, Curci C, Picerno A (2021). PMMA-based continuous hemofiltration modulated complement activation and renal dysfunction in lps-induced acute kidney injury. Front Immunol.

[27] Castellano G, Stasi A, Franzin R, Sallustio F, Divella C, Spinelli A (2019). LPS-binding protein modulates acute renal fibrosis by inducing pericyte-to-myofibroblast trans-differentiation through TLR-4 signaling. Int J Mol Sci..

[28] Curci C, Castellano G, Stasi A, Divella C, Loverre A, Gigante M (2014). Endothelial-to-mesenchymal transition and renal fibrosis in ischaemia/reperfusion injury are mediated by complement anaphylatoxins and Akt pathway. Nephrol Dial Transplant.

[29] Sallustio F, Stasi A, Curci C, Divella C, Picerno A, Franzin R (2019). Renal progenitor cells revert LPS-induced endothelial-to-mesenchymal transition by secreting CXCL6, SAA4, and BPIFA2 antiseptic peptides. FASEB J.

[30] Castellano G, Intini A, Stasi A, Divella C, Gigante M, Pontrelli P (2016). Complement modulation of anti-aging factor klotho in ischemia/reperfusion injury and delayed graft function. Am J Transplant.

[31] Harada K, Ohira S, Isse K, Ozaki S, Zen Y, Sato Y (2003). Lipopolysaccharide activates nuclear factor-kappaB through toll-like receptors and related molecules in cultured biliary epithelial cells. Lab Invest.

[32] Ciordia S, Alvarez-Sola G, Rullán M, Urman JM, Ávila MA, Corrales FJ (2021). Digging deeper into bile proteome. J Proteomics.

[33] Ciordia S, Alvarez-Sola G, Rullán M, Urman JM, Ávila MA, Corrales FJ (2022). Bile processing protocol for improved proteomic analysis. Methods Mol Biol.

[34] Castellano G, Melchiorre R, Loverre A, Ditonno P, Montinaro V, Rossini M (2010). Therapeutic targeting of classical and lectin pathways of complement protects from ischemia-reperfusion-induced renal damage. Am J Pathol.

[35] Baranova IN, Souza AC, Bocharov AV, Vishnyakova TG, Hu X, Vaisman BL (2016). Human SR-BI and SR-BII potentiate lipopolysaccharide-induced inflammation and acute liver and kidney injury in mice. J Immunol.

[36] Sallustio F, Curci C, Chaoul N, Fontò G, Lauriero G, Picerno A (2021). High levels of gut-homing immunoglobulin A+ B lymphocytes support the pathogenic role of intestinal mucosal hyperresponsiveness in immunoglobulin A nephropathy patients. Nephrol Dial Transplant.

[37] Singer M, Deutschman CS, Seymour CW, Shankar-Hari M, Annane D, Bauer M (2016). The third international consensus definitions for sepsis and septic shock (Sepsis-3). JAMA.

[38] Nicholls SJ, Andrews J, Kastelein JJP, Merkely B, Nissen SE, Ray KK (2018). Effect of serial infusions of CER-001, a pre-β high-density lipoprotein mimetic, on coronary atherosclerosis in patients following acute coronary syndromes in the CER-001 atherosclerosis regression acute coronary syndrome trial: a randomized clinical trial. JAMA Cardiol.

[39] Tardif JC, Ballantyne CM, Barter P, Dasseux JL, Fayad ZA, Guertin MC (2014). Effects of the high-density lipoprotein mimetic agent CER-001 on coronary atherosclerosis in patients with acute coronary syndromes: a randomized trial. Eur Heart J.

[40] Kidney Disease Improving Global Outcomes (KDIGO) Acute Kidney Injury Work Group (2012). KDIGO clinical practice guideline for acute kidney injury. Kidney Intern Suppl..

[41] Zarbock A, Koyner JL, Gomez H, Pickkers P, Forni L, ADQI group. Sepsis-associated acute kidney injury - treatment standard. Nephrol Dial Transplant. 2023:gfad142. Epub ahead of print. 10.1093/ndt/gfad142 37401137

[42] Dartiguelongue JB (2020). Systemic inflammation and sepsis. Part I: storm formation. Arch Argent Pediatr..

[43] Stasi A, Franzin R, Fiorentino M, Squiccimarro E, Castellano G, Gesualdo L (2021). Multifaced Roles of HDL in Sepsis and SARS-CoV-2 Infection: Renal Implications. Int J Mol Sci..

[44] Gordon BR (2004). Poor outcomes associated with low lipid and lipoprotein levels. Crit Care Med.

[45] Gordts SC, Singh N, Muthuramu I, De Geest B (2014). Pleiotropic effects of HDL: towards new therapeutic areas for HDL-targeted interventions. Curr Mol Med.

[46] Tardy C, Goffinet M, Boubekeur N, Ackermann R, Sy G, Bluteau A (2014). CER-001, a HDL-mimetic, stimulates the reverse lipid transport and atherosclerosis regression in high cholesterol diet-fed LDL-receptor deficient mice. Atherosclerosis.

[47] de Pablo R, Monserrat J, Reyes E, Díaz D, Rodríguez-Zapata M, de la Hera A (2013). Circulating sICAM-1 and sE-Selectin as biomarker of infection and prognosis in patients with systemic inflammatory response syndrome. Eur J Intern Med.

[48] Qin Q, Liang L, Xia Y (2021). Diagnostic and prognostic predictive values of circulating sTREM-1 in sepsis: a meta-analysis. Infect Genet Evol.

[49] Jolly L, Carrasco K, Salcedo-Magguilli M, Garaud JJ, Lambden S, van der Poll T (2021). sTREM-1 is a specific biomarker of TREM-1 pathway activation. Cell Mol Immunol.

[50] Pool R, Gomez H, Kellum JA (2018). Mechanisms of organ dysfunction in sepsis. Crit Care Clin.

[51] Tanaka S, De Tymowski C, Stern J, Bouzid D, Zappella N, Snauwaert A (2022). Relationship between liver dysfunction, lipoprotein concentration and mortality during sepsis. PLoS One.

[52] Fleischmann-Struzek C, Mellhammar L, Rose N, Cassini A, Rudd KE, Schlattmann P (2020). Incidence and mortality of hospital- and ICU-treated sepsis: results from an updated and expanded systematic review and meta-analysis. Intensive Care Med.

[53] Rudd KE, Johnson SC, Agesa KM, Shackelford KA, Tsoi D, Kievlan DR (2020). Global, regional, and national sepsis incidence and mortality, 1990–2017: analysis for the Global Burden of Disease Study. Lancet.

