# Supplementary material for: Beneficial effects of recombinant CER-001 high-density lipoprotein infusion in sepsis: results from a bench to bedside translational research project
Source: BMC Med. 2023 Nov 2;21:392. doi: 10.1186/s12916-023-03057-5 (PMC10621167; doi:10.1186/s12916-023-03057-5)
Supplement: Supplementary file 1 — Additional file 1: Table S1. Statistical differences between Tukey correction and Uncorrected Fisher's LSD for significant comparisons in pre-clinical model. [file 12916_2023_3057_MOESM1_ESM.docx]

| **Parameter** | **Statistical Test Used** | **Data Transformation** | **Time Points** | **Significant Findings**  **(with Tukey Correction)** | **Significant Findings**  **(No Correction)** |
| --- | --- | --- | --- | --- | --- |
| Survival | Logrank test for trend | N/A | N/A | Treatment Effect p=0.0265 | |
| LPS | 2-Way ANOVA with Repeated Measures | Change from Baseline with LOCF | T0, T1, T3, T6 and T24 | Overall Treatment Effect p=0.0029  T1 LPS vs CER20 p=0.0462  T1 LPS vs CER20x2 p=0.0133  T6 LPS vs CER20x2 p=0.0015  *T6 CER20 vs CER20x2 None*  T24 LPS vs CER20x2 p=0.0016 | Overall Treatment Effect p=0.0029  T1 LPS vs CER20 p=0.0193  T1 LPS vs CER20x2 p=0.0058  T6 LPS vs CER20x2 p=0.0006  *T6 CER20 vs CER20x2 p=0.0303*  T24 LPS vs CER20x2 p=0.0007 |
| IL-6 |  |  |  | Overall Treatment Effect p=0.0021  T1 LPS vs CER20 p=0.0168  T1 LPS vs CER20x2 p=0.0179  *T3 LPS vs CER20 None*  T6 LPS vs CER20x2 p=0.0087  T24 LPS vs CER20x2 p=0.0086 | Overall Treatment Effect p=0.0021  T1 LPS vs CER20 p=0.0073  T1 LPS vs CER20x2 p=0.0078  *T3 LPS vs CER20 p=0.0282*  T6 LPS vs CER20x2 p=0.0038  T24 LPS vs CER20x2 p=0.0037 |
| TNF-α |  |  |  | Overall Treatment Effect p<0.0001  T3 LPS vs CER20 p=0.0020  T3 LPS vs CER20x2 p=0.0026  T6 LPS vs CER20 p=0.0048  T6 LPS vs CER20x2 p=0.0010  T6 CER20 vs CER20x2 p=0.0255  T24 LPS vs CER20 p=0.0004  T24 LPS vs CER20x2 p<0.0001 | Overall Treatment Effect p<0.0001  T3 LPS vs CER20 p=0.0008  T3 LPS vs CER20x2 p=0.0010  T6 LPS vs CER20 p=0.0019  T6 LPS vs CER20x2 p=0.0004  T6 CER20 vs CER20x2 p=0.0104  T24 LPS vs CER20 p=0.0001  T24 LPS vs CER20x2 p<0.0001 |
| MCP-1 |  |  |  | Overall Treatment Effect p=0.0113  T1 LPS vs CER20 p=0.0219  T1 LPS vs CER20x2 p=0.0005  T6 LPS vs CER20x2 p=0.0207  T24 LPS vs CER20x2 p=0.0009 | Overall Treatment Effect p=0.0113  T1 LPS vs CER20 p=0.0092  T1 LPS vs CER20x2 p=0.0002  T6 LPS vs CER20x2 p=0.0089  T24 LPS vs CER20x2 p=0.0004 |
| VCAM |  |  |  | Overall Treatment Effect p=0.0101  *T6 LPS vs CER20x2 None*  T6 CER20 vs CER20x2 p=.0010  *T24 LPS vs CER20 None*  *T24 LPS vs CER20x2 None*  T24 CER20 vs CER20x2 p=0.0248 | Overall Treatment Effect p=0.0101  *T6 LPS vs CER20x2 p=0.0358*  T6 CER20 vs CER20x2 p=.0004  *T24 LPS vs CER20 p=0.0416*  *T24 LPS vs CER20x2 p=0.0238*  T24 CER20 vs CER20x2 p=0.0107 |
| ICAM |  |  |  | *T3 CER20 vs CER20x2 None*  T24 LPS vs CER20x2 p=0.0251 | *T3 CER20 vs CER20x2 p=0.0387*  T24 LPS vs CER20x2 p=0.0108 |
| ALT |  |  |  | Overall Treatment Effect p=0.0306  *T3 LPS vs CER20x2 None*  *T6 LPS vs CER20x2 None*  T24 LPS vs CER20 p=0.0409  T24 LPS vs CER20x2 p=0.0098 | Overall Treatment Effect p=0.0306  *T3 LPS vs CER20x2 p=0.0463*  *T6 LPS vs CER20x2 p=0.0471*  T24 LPS vs CER20 p=0.0173  T24 LPS vs CER20x2 p=0.0043 |
| Comp Classic |  |  | T0, T1, T3 and T24 | Overall Treatment Effect p<0.0001  T1 LPS vs CER20 p=0.0030  T1 LPS vs CER20x2 p=0.0061  T3 LPS vs CER20 p=0.0026  T3 LPS vs CER20x2 p=0,0044  T24 LPS vs CER20 p=0.0017  T24 LPS vs CER20x2 p=0.0022 | Overall Treatment Effect p<0.0001  T1 LPS vs CER20 p=0.0012  T1 LPS vs CER20x2 p=0.0026  T3 LPS vs CER20 p=0.0011  T3 LPS vs CER20x2 p=0,0017  T24 LPS vs CER20 p=0.0007  T24 LPS vs CER20x2 p=0.0009 |
| Comp Alternate |  |  |  | Overall Treatment Effect p<0.0001  T1 LPS vs CER20 p=0.0004  T1 LPS vs CER20x2 p=0.0002  T1 CER20 vs CER20x2 p=0.0046  T3 LPS vs CER20 p=0.0009  T3 LPS vs CER20x2 p=0.0011  T24 LPS vs CER20 p=0.0004  T24 LPS vs CER20x2 p=0.0003 | Overall Treatment Effect p<0.0001  T1 LPS vs CER20 p=0.0002  T1 LPS vs CER20x2 p<0.0001  T1 CER20 vs CER20x2 p=0.0018  T3 LPS vs CER20 p=0.0004  T3 LPS vs CER20x2 p=0.0004  T24 LPS vs CER20 p=0.0001  T24 LPS vs CER20x2 p=0.0001 |
| Comp Lectin |  |  |  | None | None |
| sCR |  |  | T0, T3 and T24 | Overall Treatment Effect p<0.0001  *T3 LPS vs CER20 None*  T3 LPS vs CER20x2 p=0.0179  T24 LPS vs CER20 p=0.0003  T24 LPS vs CER20x2 p=0.0005 | Overall Treatment Effect p<0.0001  *T3 LPS vs CER20 p=0.0274*  T3 LPS vs CER20x2 p=0.0073  T24 LPS vs CER20 p=0.0001  T24 LPS vs CER20x2 p=0.0002 |
| sKIM-1 |  |  |  | None | *T3 LPS vs CER20x2 p=0.0456* |
| sCystatin-C |  |  |  | Overall Treatment Effect p=0.0033  *T3 LPS vs CER20 None*  T3 LPS vs CER20x2 p=0.0115  *T24 LPS vs CER20 None*  T24 LPS vs CER20x2 p=0.0446 | Overall Treatment Effect p=0.0033  *T3 LPS vs CER20 p=0.0385*  T3 LPS vs CER20x2 p=0.0045  *T24 LPS vs CER20 p=0.0310*  T24 LPS vs CER20x2 p=0.0196 |
| UKIM-1/ U-Creatinine |  |  | T0 and T24 | Overall Treatment Effect p<0.0001  T24 LPS vs CER20 p<0.0001  T24 LPS vs CER20x2 p<0.0001 | Overall Treatment Effect p<0.0001  T24 LPS vs CER20 p<0.0001  T24 LPS vs CER20x2 p<0.0001 |
| uCystatin-C/ U-Creatinine |  |  |  | Overall Treatment Effect 0.0015  T24 LPS vs CER20 p<0.0010  T24 LPS vs CER20x2 p<0.0010 | Overall Treatment Effect 0.0052  T24 LPS vs CER20 p<0.0001  T24 LPS vs CER20x2 p<0.0001 |
| s APO A-I | 2-Way ANOVA with Repeated Measures | Change from Baseline with LOCF | T0, T1, T3, T4, T6 and T24 | Overall Treatment Effect p<0.0001  T1 LPS vs CER20 p= 0.0004  T1 LPS vs CER20x2 p <0.0001  T3 LPS vs CER20 p= 0.0004  T3 LPS vs CER20x2 p <0.0001  T4 LPS vs CER20 p <0.0001  T4 LPS vs CER20x2 p <0.0001  T4 CER20 vs CER20x2 p<0.0001  T6 LPS vs CER20 p <0.0001  T6 LPS vs CER20x2 p <0.0001  T6 CER20 vs CER20x2 p=0.0001 | Overall Treatment Effect p<0.0001  T1 LPS vs CER20 p= 0.0002  T1 LPS vs CER20x2 p <0.0001  T3 LPS vs CER20 p= 0.0002  T3 LPS vs CER20x2 p <0.0001  T4 LPS vs CER20 p <0.0001  T4 LPS vs CER20x2 p <0.0001  T4 CER20 vs CER20x2 p<0.0001  T6 LPS vs CER20 p <0.0001  T6 LPS vs CER20x2 p <0.0001  T6 CER20 vs CER20x2 p<0.0001 |
| APO A-I bile | 1-Way ANOVA | None | N/A | Overall Treatment Effect p<0.0001  LPS vs CER20 p= 0.04  LPS vs CER20x2 p=0.0018  CER20 vs CER20x2 p=0.0006 | Overall Treatment Effect p <0.0001  LPS vs CER20 p= 0.0176  LPS vs CER20x2 p <0.0001  CER20 vs CER20x2 p= 0.0002 |
| LPS bile | 1-Way ANOVA | None | N/A | Overall Treatment Effect p=0.0016  LPS vs CER20x2 p<0.0001  CER20 vs CER20x2 p=0.0052 | Overall Treatment Effect p=0.0016  LPS vs CER20x2 p=0.00007  CER20 vs CER20x2 p=0.0022 |
| LPS liver (WB) | 1-Way ANOVA | None | N/A | Overall Treatment Effect p<0.0001  LPS vs CER20 p<0.0001  LPS vs CER20x2 p<0.0001 | Overall Treatment Effect p<0.0001  LPS vs CER20 p<0.0001  LPS vs CER20x2 p<0.0001 |
| Urine Output | 1-Way ANOVA | None | N/A | Overall Treatment Effect p=0.0385  LPS vs CER20x2 p=0.0380 | Overall Treatment Effect p=0.0385  LPS vs CER20x2 p=0.0380 |
| Tubular Injury Score |  |  |  | Overall Treatment Effect p<0.0001  LPS vs CER20 p=0.004  LPS vs CER20x2 p<0.0001  CER20 vs CER20x2 p<0.0001 | Overall Treatment Effect p<0.0001  LPS vs CER20 p=0.004  LPS vs CER20x2 p<0.0001  CER20 vs CER20x2 p<0.0001 |
| Glomerular Injury Score |  |  |  | Overall Treatment Effect p<0.0001  LPS vs CER20 p=0.073  LPS vs CER20x2 p<0.0001  CER20 vs CER20x2 p=0.0205 | Overall Treatment Effect p<0.0001  LPS vs CER20 p=0.073  LPS vs CER20x2 p<0.0001  CER20 vs CER20x2 p=0.0205 |
| Hepatic Injury Score |  |  |  | Overall Treatment Effect p<0.0001  LPS vs CER20 p=0.017  LPS vs CER20x2 p<0.0001  CER20 vs CER20x2 p=0.0152 | Overall Treatment Effect p<0.0001  LPS vs CER20 p=0.017  LPS vs CER20x2 p<0.0001  CER20 vs CER20x2 p=0.0152 |
