# Supplementary figures and images for: Beneficial effects of recombinant CER-001 high-density lipoprotein infusion in sepsis: results from a bench to bedside translational research project
Source: BMC Med. 2023 Nov 2;21:392. doi: 10.1186/s12916-023-03057-5 (PMC10621167; doi:10.1186/s12916-023-03057-5)

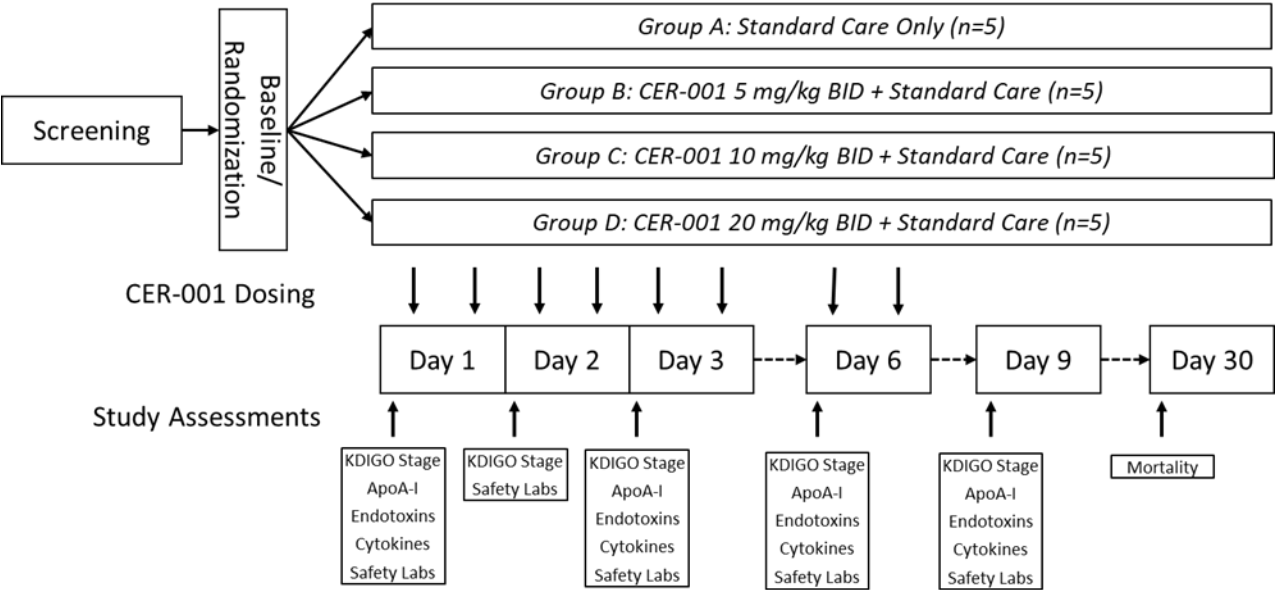

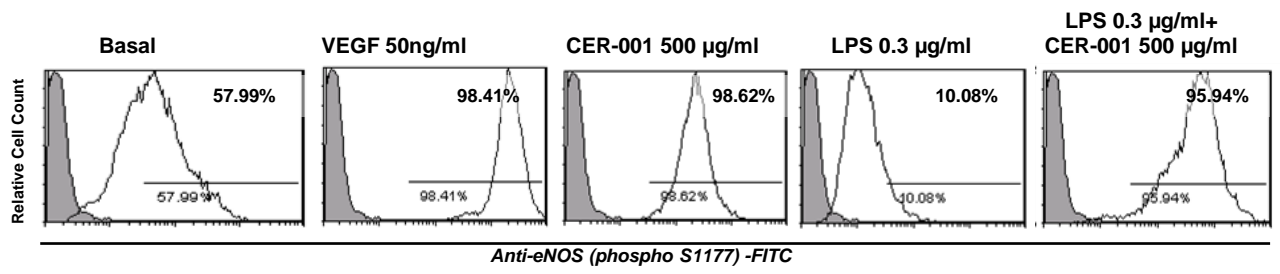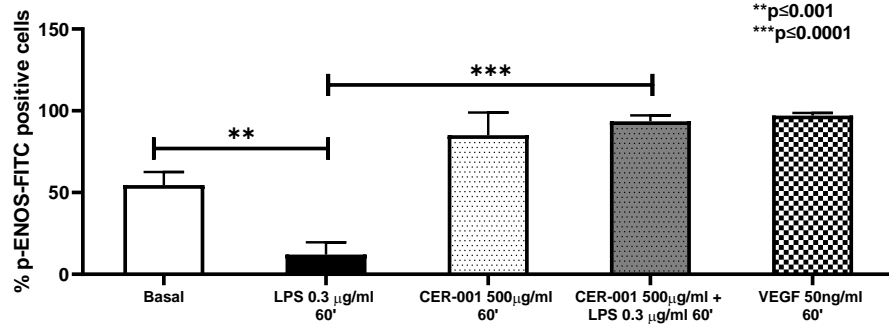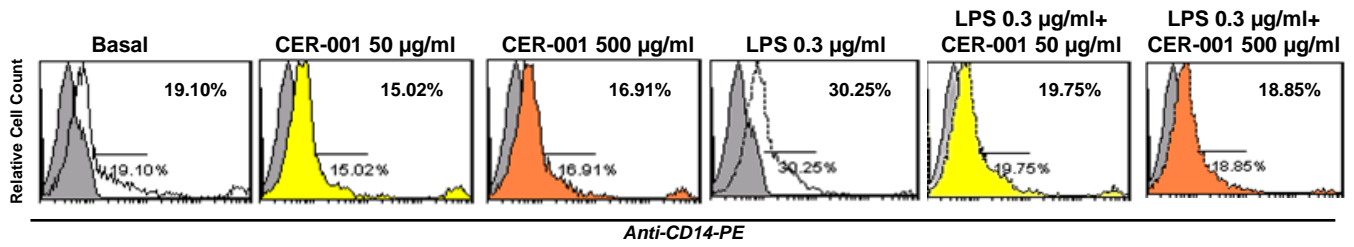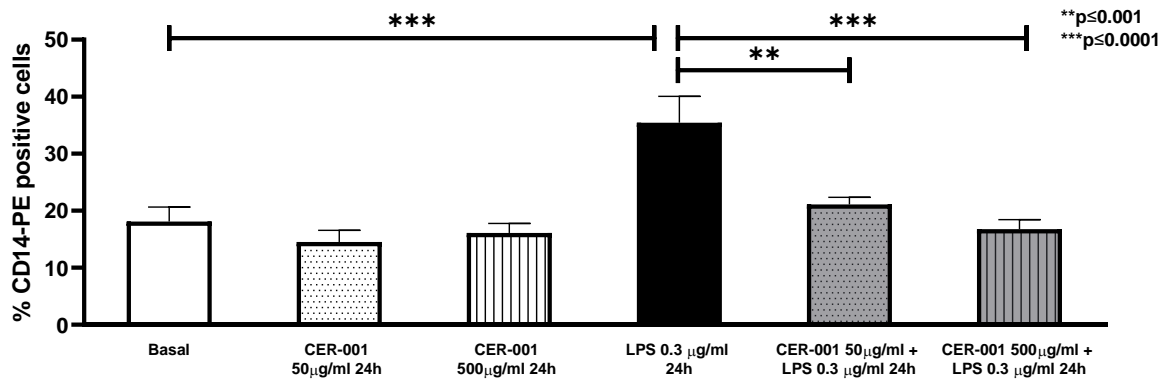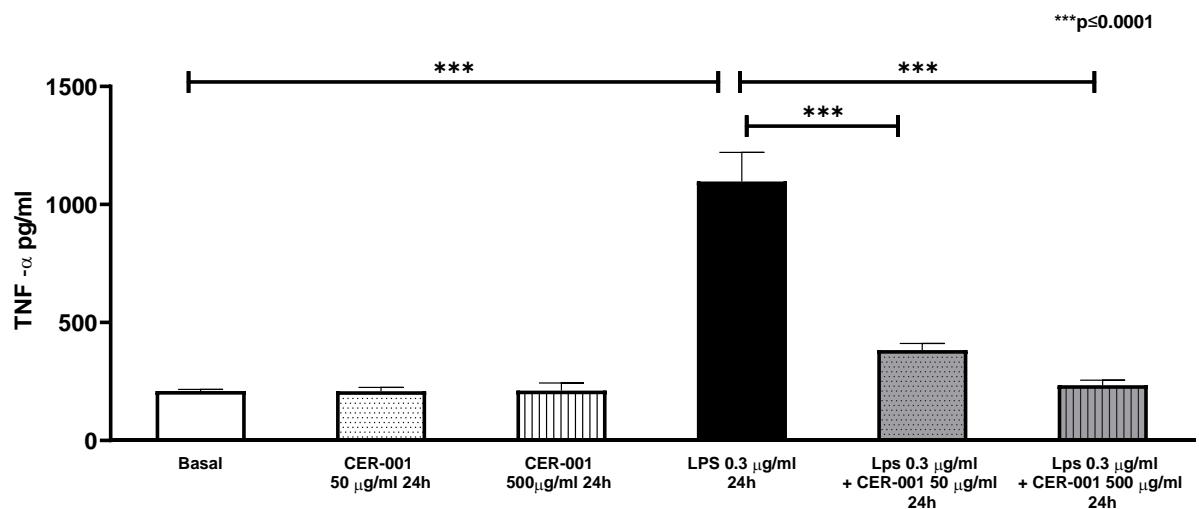

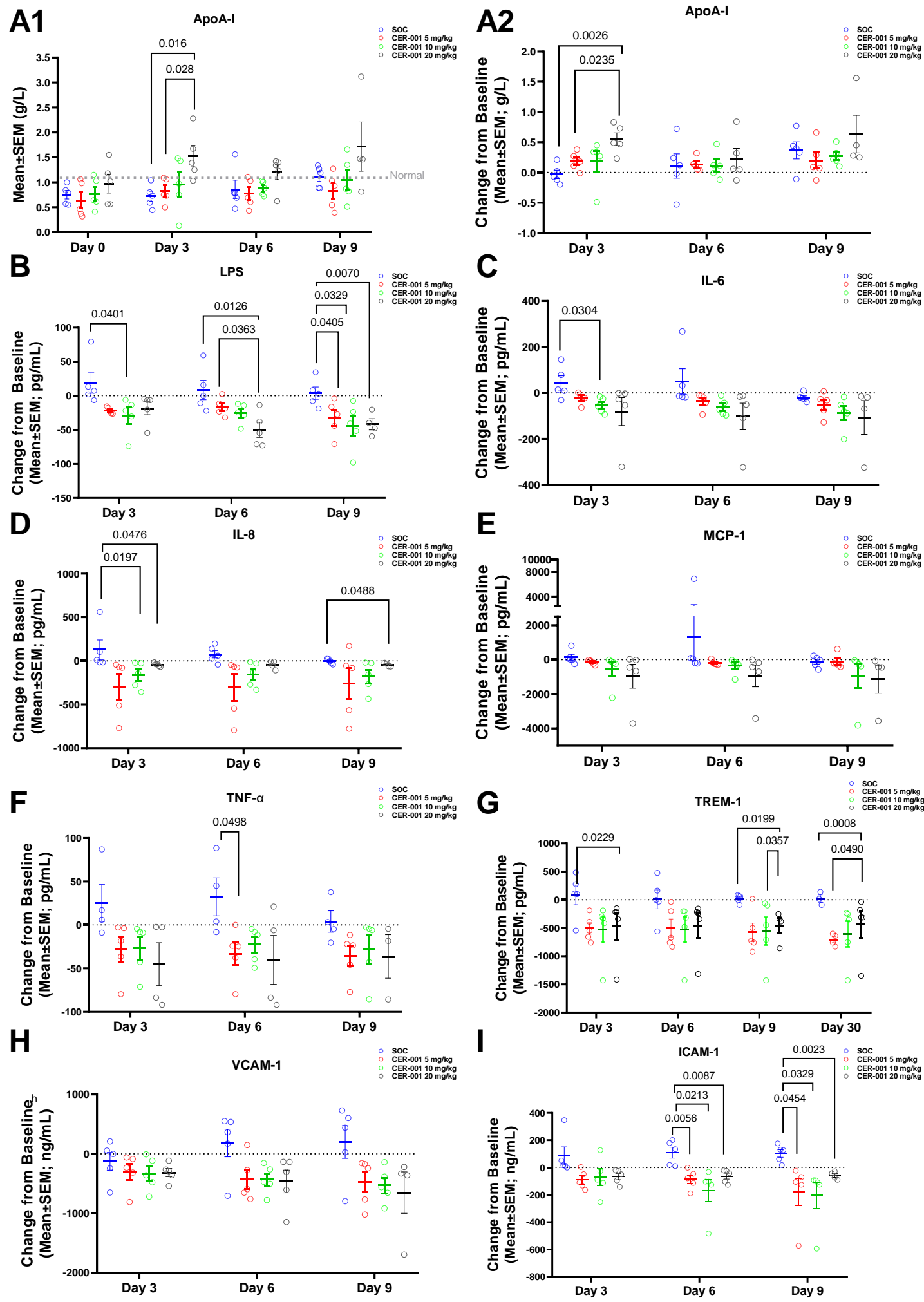

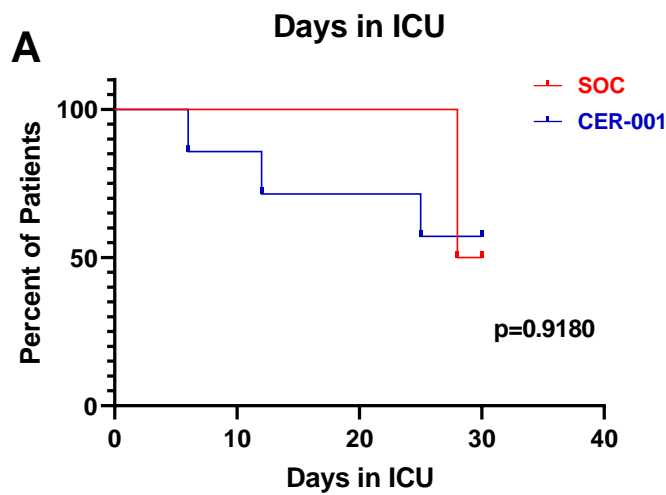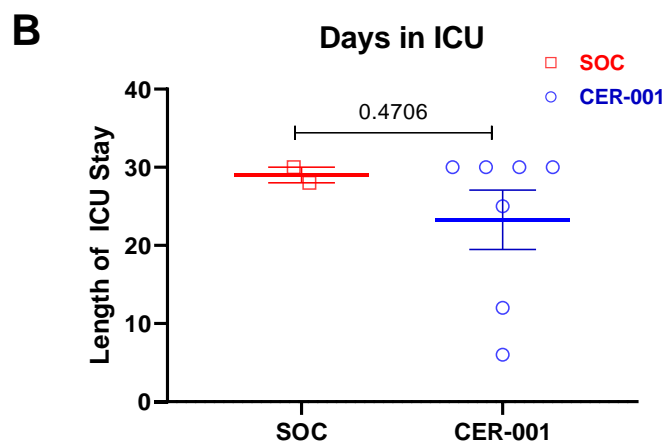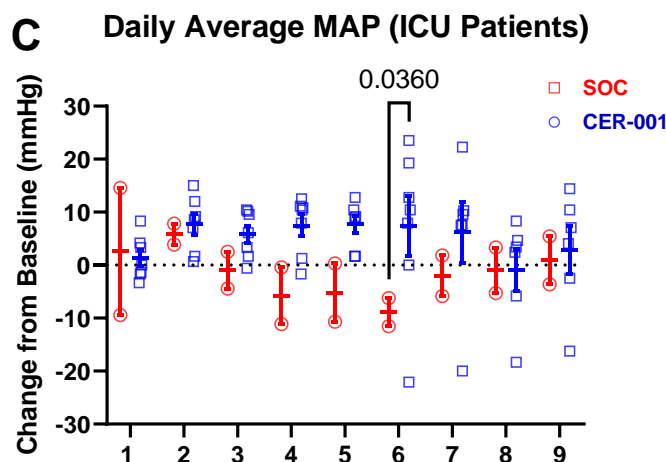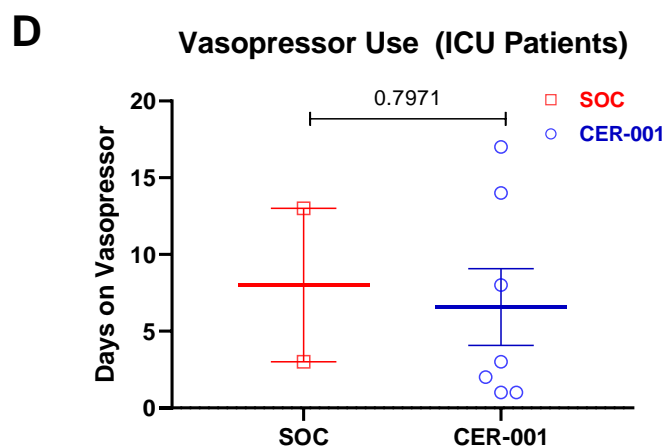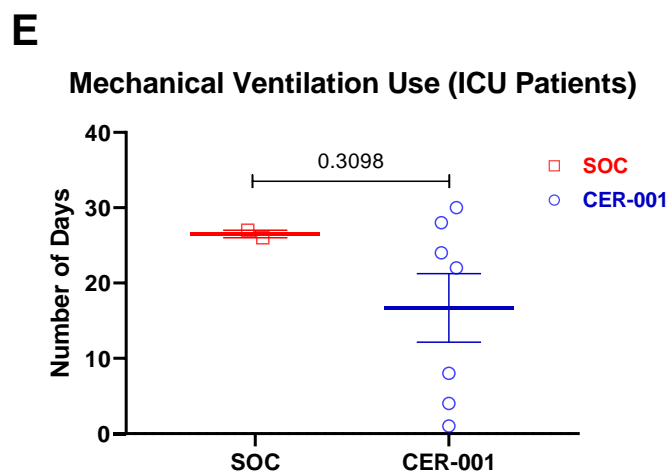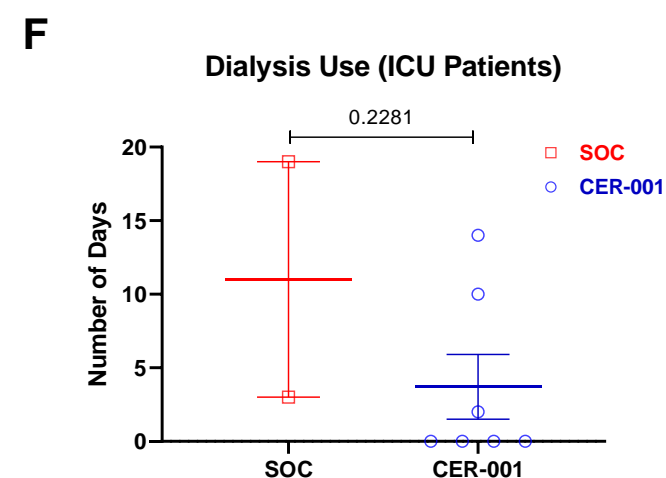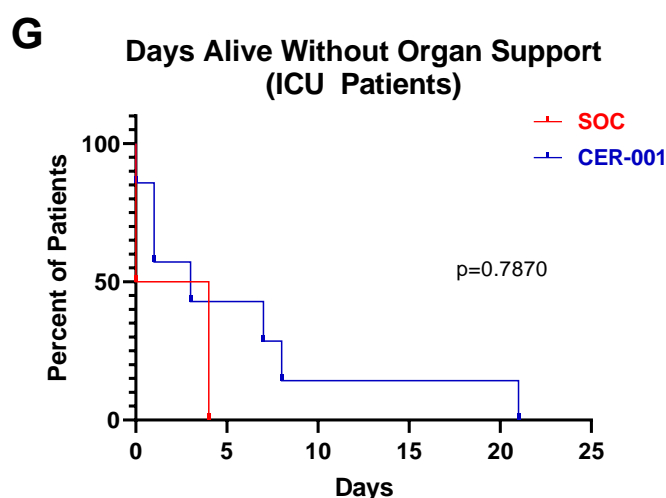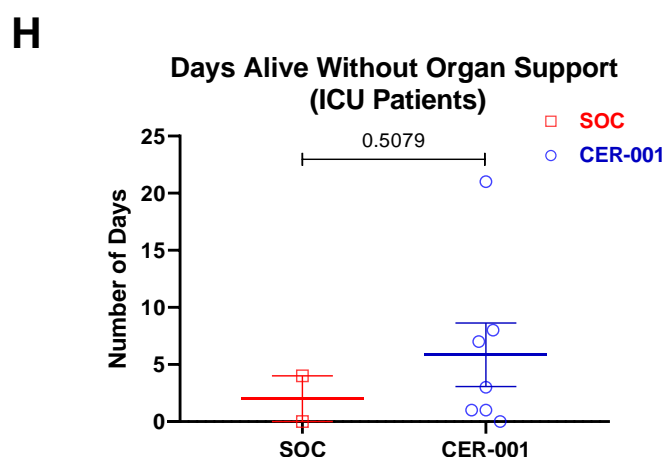

Supplement: Supplementary file 2 — Additional file 2: Figure S1. RACERS Trial design. Figure S2. In vitro effects of CER-001 on LPS-treated endothelial cells and PBMCs. A-B. Cultured endothelial cells were stimulated with LPS at 0.3 ug/ml and/or CER-001 at 50 and 500 ug/ml for 60. FACS (Fluorescence Activated Cell Sorting) showed a strong decrease of eNOS (phospho S1177) (p-ENOS) after 60’ of LPS stimulation compared to basal and VEGF as a positive control. CER-001 supplementation at 500 ug/ml, completely reversed LPS effects. A. Representative data from one out of a total of three experiments are shown. B. Histograms indicate p-ENOS expression levels. C-E. PBMC from 3 different healthy donors were stimulated with LPS at 0.3 ug/ml and/or CER-001 at 50 and 500 ug/ml for 24 h. C-D FACS showed a strong upregulation of CD14 surface expression 24 h following LPS stimulation. PBMCs treated with LPS and CER-001 in combination maintained CD14 expression at basal level. C. One representative of three independent experiments is shown. D. Histograms indicate CD14 expression levels. E. PBMC culture supernatants were analyzed by ELISA. After 24 h from LPS stimulation, PBMCs increased TNF-α synthesis. Stimulation of PBMCs with CER-001 at 50 and 500 ug/ml alone did not influence TNF-α production. The addition of CER-001, both at 50 and 500 ug/ml, in culture media of LPS-activated PBMCs reverted LPS effects. A-E Data are representative of three independent experiments. Data are shown as mean ± standard deviation (SD) and compared with the Student-t test. Figure S3. Effect of CER-001 on ApoA-I level, LPS removal and inflammatory response in the pilot study RACERS presented by individual treatment groups. A-I. Serum levels of ApoA-I, LPS, IL-6, IL-8, MCP1, TNF-α, sTREM-1, VCAM and ICAM were measured using ELISA kits. Data for panels A2-I are represented as changes from baseline (Day 1 pre-dose) values. Treatment differences were assessed by using a mixed model ANOVA for values on Days 3, 6 and 9 with a significance le [file 12916_2023_3057_MOESM2_ESM.pdf]

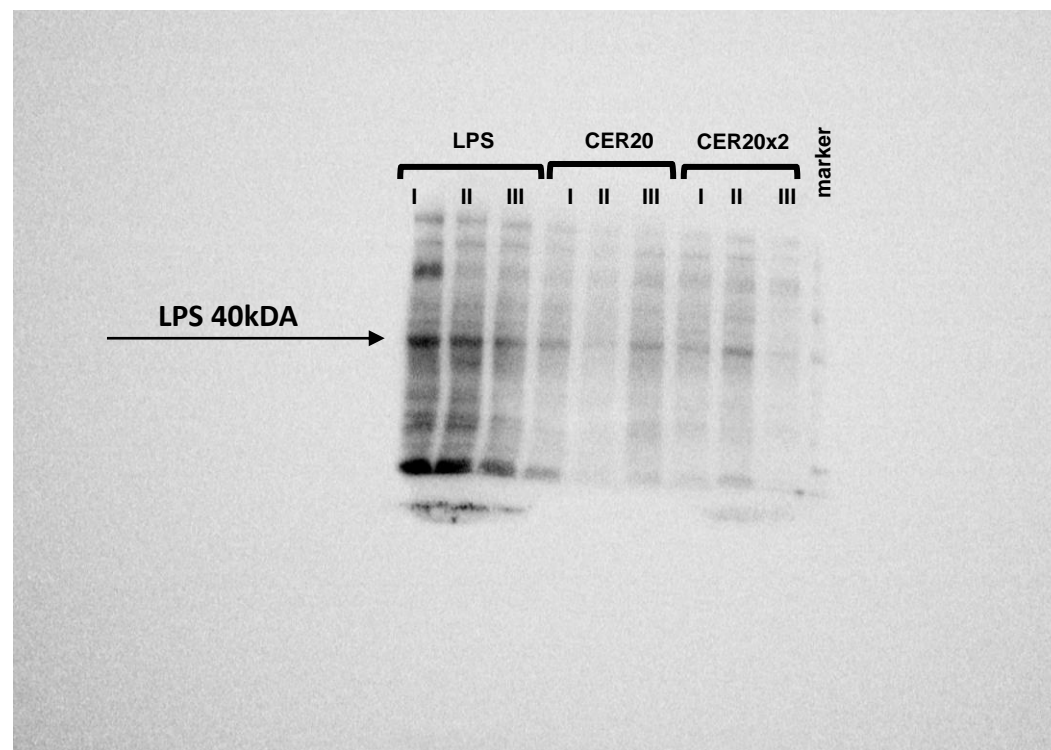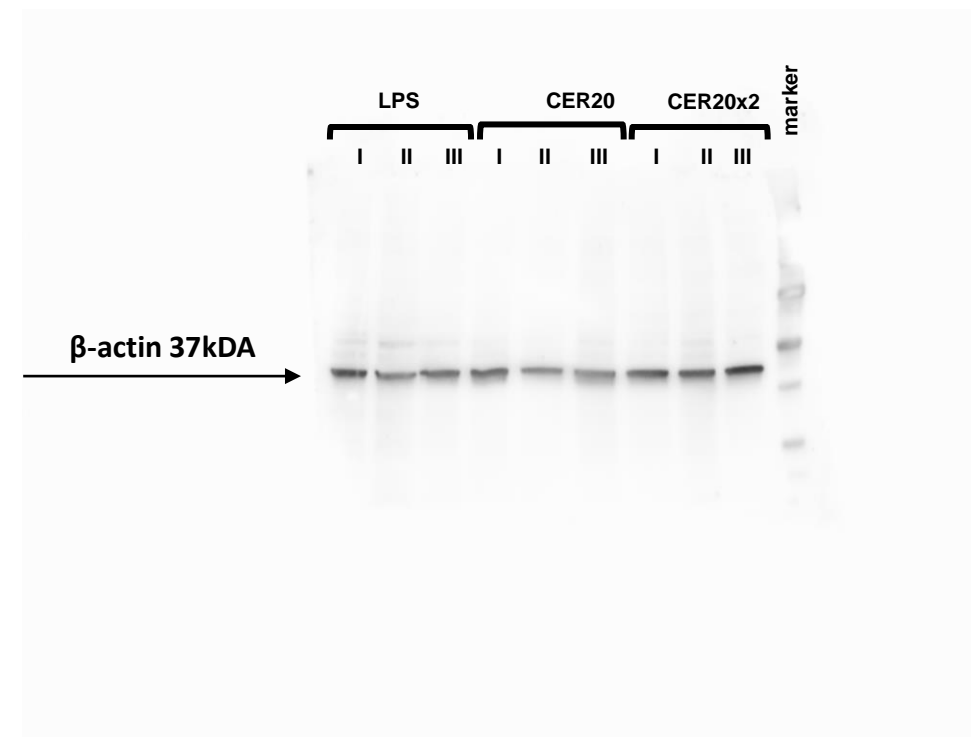

Supplement: Supplementary file 5 — Additional file 5. [file 12916_2023_3057_MOESM5_ESM.pdf]
