# Supplementary material for: Beneficial effects of recombinant CER-001 high-density lipoprotein infusion in sepsis: results from a bench to bedside translational research project
Source: BMC Med. 2023 Nov 2;21:392. doi: 10.1186/s12916-023-03057-5 (PMC10621167; doi:10.1186/s12916-023-03057-5)
Supplement: Supplementary file 3 — Additional file 3: Table S2. Adverse events among participants randomized reported in the pilot clinical study. [file 12916_2023_3057_MOESM3_ESM.docx]

**Supplementary Table 2. Adverse events in the RACERS study.**

| **Pt #** | **Adverse Event**  **(Verbatim Term)** | | **AE Onset** | **AE Stop** | **Grade** | **Outcome** | **Related to Study Drug** | **Action** | | | **SAE?** | |
| --- | --- | --- | --- | --- | --- | --- | --- | --- | --- | --- | --- | --- |
| **Standard of Care** | | | | | | | | | | | | |
| 12 | Septic shock due to secondary fungal infection | | Day 24 | Day 27 | 4 | Fatal | Def No | NA | | | Yes | |
| **CER-001 5 mg/kg BID** | | | | | | | | | | | |  |
| 1 | Anastomotic leak and surgical wound dehiscence | | Day 8 | Day 11 | 4 | Fatal | Def No | NA | | Yes | |  |
| 1 | Hypertriglyceridemia | | Day 3 | Day 9 | 1 | Recovered | Probably | None | | No | |  |
| 7 | Gastric suture dehiscence and placement of gastric endoprosthesis | | Day 6 | Day 6 | 2 | Recovered | Def No | None | | No | |  |
| 9 | Atrial fibrillation | | Day 3 | Day 4 | 3 | Recovered | Prob No | DC | | Yes | |  |
| 17 | Hypertriglyceridemia | | Day 3 | Day 9 | 1 | Recovered | Probably | None | | No | |  |
| 17 | Covid-19 infection | | Day 17 | Day 30 | 1 | Recovered | Def No | NA | | No | |  |
| **CER-001 10 mg/kg BID** | | | | | | | | | | | |  |
| 3 | Hypertriglyceridemia | | Day 3 | Day 41 | 1 | Recovered | Probably | None | | No | |  |
| 8 | Sepsis and acute kidney injury due to pneumonia | | Day 27 | Day 35 | 2 | Recovered | Def No | NA | | No | |  |
| 10 | Chest pressure | | Day 2 | Day 3 | 2 | Recovered | Prob No | DC | No | | |  |
| 13 | Platelet count reduction | | Day 2 | Ongoing | 2 | NR | Prob No |  | | No | |  |
| 13 | Bloodstream infection due to Candida Parapsilosis | | Day 5 | Ongoing | 1 | NR | Prob No |  | | No | |  |
| 13 | Bloodstream infection due to Acinetobacter Baumanii | | Day 10 | Day 29 | 2 | Recovered | Prob No |  | | No | |  |
| 19 | Bloodstream infection due to enterococcus faecalis | | Day 13 | Day 32 | 2 | Recovered | Def No |  | | No | |  |
| 19 | Ventilator associated pneumonia due to Acinetobacter Baumanii | | Day 21 | Day 48 | 2 | Rec Seq | Def No |  | | No | |  |
| **CER-001 20 mg/kg BID** | | | | | | | | | | | |  |
| 2 | Creatinine increase | | Day 32 | Ongoing | 2 | NR | Def No |  | | No | |  |
| 2 | Hypertriglyceridemia | | Day 3 | Day 6 | 1 | Recovered | Probably |  | | No | |  |
| 5 | Hypertriglyceridemia | | Day 3 | Day 35 | 1 | Recovered | Probably |  | | No | |  |
| 5 | Hypertension | | Day 3 | Ongoing | 1 | NR | Def No |  | | No | |  |
| 11 | Hypertriglyceridemia | | Day 3 | Day 43 | 1 | Recovered | Probably |  | | No | |  |
| 14 | Hypertriglyceridemia | | Day 3 | Day 6 | 1 | Recovered | Probably |  | | No | |  |
| Abbreviations: | | Grade – 1=Mild; 2=Moderate; 3=Severe; 4=Life-threatening  Outcome – Rec=Recovered; Rec Seq=Recovered with Sequelae; NR=Not Recovered/Not Resolved  Related to Study Drug – Def No=Definitely Not; Prob No=Probably Not  Action– DC=Study Drug Discontinued; NA=Not applicable (treatment already completed) | | | | | | | | | |  |
