# Supplementary material for: Beneficial effects of recombinant CER-001 high-density lipoprotein infusion in sepsis: results from a bench to bedside translational research project
Source: BMC Med. 2023 Nov 2;21:392. doi: 10.1186/s12916-023-03057-5 (PMC10621167; doi:10.1186/s12916-023-03057-5)
Supplement: Supplementary file 4 — Additional file 4: Table S3. Main clinical outcomes at 30 days of ICU patients of RACERS participants. [file 12916_2023_3057_MOESM4_ESM.docx]

**Supplementary Table 3.** Main clinical outcomes at 30 days of ICU patients of RACERS participants

| **Intervention** | **SOC group** | **CER-001 group** | **p-value** |
| --- | --- | --- | --- |
|  | **n=2** | **n=7** |  |
| Duration of mechanical ventilation (days), mean (SEM) | 26.5 (0.5) | 17.1 (4.3) | 0.309 |
| Days on vasopressors, mean (SEM) | 8 (5) | 6.5 (2.5) | 0.797 |
| Renal Replacement Therapy (RRT), n (%) | 2 (100%) | 3 (42.8%) | 0.151 |
| Days on RRT, mean (SEM) | 11 (8) | 3.7 (2.2) | 0.228 |
| Days alive without organ support*, mean (SEM) | 2 (2) | 5.9 (2.7) | 0.507 |
| ICU stay (days), mean (SEM) | 28.5 (1.5) | 23.2 (3.6) | 0.470 |
| Hospital stay (days), mean (SEM) | 28.5 (1.5) | 27.5 (2.4) | 0.852 |
| Mortality at 30 days, n (%) | 1 (50%) | 1 (14.2%) | 0.389 |

**Legend**. SEM Standard Error for the Mean; ICU Intensive Care Unit; RRT Renal Replacement Therapy

*number of days without need for dialysis, mechanical ventilation and vasopressors
